# Effectiveness of COVID-19 vaccines against Omicron and Delta hospitalisation, a test negative case-control study

**DOI:** 10.1038/s41467-022-33378-7

**Published:** 2022-09-30

**Authors:** Julia Stowe, Nick Andrews, Freja Kirsebom, Mary Ramsay, Jamie Lopez Bernal

**Affiliations:** 1 UK Health Security Agency, London, UK; 2 NIHR Health Protection Research Unit in Vaccines and Immunisation, London School of Hygiene and Tropical Medicine, London, UK; 3 NIHR Health Protection Research Unit in Respiratory Infections, Imperial College London, London, UK

**Keywords:** Infectious diseases, Statistics, SARS-CoV-2, Vaccines

## Abstract

The Omicron variant has been associated with reduced vaccine effectiveness (VE) against mild disease with rapid waning. Meanwhile Omicron has also been associated with milder disease. Protection against severe disease has been substantially higher than protection against infection with previous variants. We used a test-negative case-control design to estimate VE against hospitalisation with the Omicron and Delta variants using PCR testing linked to hospital records. We investigated the impact of increasing the specificity and severity of hospitalisation definitions on VE. Among 18–64-year-olds using cases admitted via emergency care, VE after a 3rd dose peaked at 82.4% and dropped to 53.6% by 15+ weeks after the 3rd dose; using all admissions for > = 2 days stay with a respiratory code in the primary diagnostic field VE ranged from 90.9% to 67.4%; further restricting to those on oxygen/ventilated/intensive care VE ranged from 97.1% to 75.9%. Among 65+ year olds the equivalent VE estimates were 92.4% to 76.9%; 91.3% to 85.3% and 95.8% to 86.8%. Here we show that with milder Omicron disease contamination of hospitalisations with incidental cases is likely to reduce VE estimates. VE estimates increase, and waning is reduced, when specific hospitalisation definitions are used.

## Abstract (web summary)

SARS-CoV-2 variants of concern have been associated with reduced vaccine effectiveness, even after a booster dose. In this study, authors aim to estimate vaccine effectiveness against hospitalisation with the Omicron and Delta variants, using different definitions of hospitalisation in secondary care data.

## Introduction

There has been a global increase in COVID-19 cases associated with the Omicron variant between November 2021 and March 2022^1^. Nevertheless, surges in severe disease, as indicated by hospitalisations, ICU admissions or deaths, have not matched those of previous waves of the pandemic^2^. A range of factors are likely to contribute to this divergence, including lower inherent severity of Omicron compared to previous variants, a greater proportion of the population with immunity from vaccination and/or prior infection, and sustained protection against severe disease^3,4^.

Early data indicated a reduced neutralising antibody response to the Omicron variant^5–7^. Real-world studies have since found reduced effectiveness of COVID-19 vaccines against infection or mild disease with the Omicron variant^8–10^. Receipt of a 3rd dose improves protection; however, this appears to wane rapidly from the second month after vaccination^8^. Evidence on protection against severe disease is mixed with some studies suggesting substantially reduced effectiveness against hospitalisation compared to the Delta variant even with 3rd doses^11,12^, whereas other studies suggest very high levels of effectiveness of over 90%^9,13,14^. There is currently limited data on the duration of protection against severe disease.

In this study, we assess the effectiveness of COVID-19 vaccines against hospitalisation in those testing positive by PCR for Omicron and Delta variants. In the past, we have done this using symptomatic community tested cases subsequently hospitalised through emergency care for a non-accident reason within 2 weeks of their positive test with a test-negative case–control (TNCC) design^15^. This has yielded estimates of the effectiveness of over 90% against Alpha and Delta variants. However, given that all individuals who are hospitalised for any reason in the UK are tested for COVID-19, and with the lower severity of Omicron and the high incidence, an increasing proportion of those hospitalised who also test positive may be hospitalised with COVID-19 as an incidental finding rather than hospitalised as a result of COVID-19. This would lead to underestimation of effectiveness against hospitalisation because the “with COVID-19” cases would be expected to have effectiveness similar to that seen against infection. To investigate this specificity of outcome issue we have obtained data on coded hospital discharges in those PCR tested, including on primary diagnosis, length of stay, oxygen use, ventilation and admission to intensive care. Whilst these data are not as timely as using emergency care admission data, they allow the identification of those more likely to have been admitted due to COVID-19.

## Results

### Descriptive characteristics

After linkage of testing data to hospitalised cases in ECDS or a respiratory coded SUS episode and to the NIMS vaccination database, and selection of the Delta and Omicron assigned cases and the controls, the total number of tests in the study period was 409,985 of which 115,720 were cases and 294,265 controls. A total of 51,115 (44.2%) of these cases and 34,556 (11.7%) of these controls had a Pillar 2 test as the earliest test and of these 38,150 cases and 31,552 controls were symptomatic and included in the ECDS analysis. For the ECDS analyses where all symptomatic controls were used irrespective of hospitalisation the total number of controls included was 6,759,286 whilst the analysis to assess symptomatic vaccine effectiveness using the Pillar 2 data included 27,256 cases along with these controls.

The characteristics of hospitalised cases and controls for the Omicron and Delta period analyses are shown in Supplementary Table S1 (age 18–64) and Supplementary Table S2 (age 65 years and over). Note that some controls contribute to both the Omicron and Delta analyses. Pillar 2 symptomatic ECDS admissions in cases are much lower than SUS admissions for those aged over 65, even when restricting to those with a 2-day stay and primary diagnostic field coded. This difference is less for age 18–64 and for Omicron (18–64). Of the SUS admissions the proportion with a recorded intervention (oxygen/ventilation/ICU) is significantly higher (Chi-squared *P* < 0.001) for Delta cases (age 18–64: 20.8%; age 65+: 21.2%) than Omicron cases (age 18–64: 2.5%; age 65+: 6.6%) and higher for cases than controls except for Omicron cases (2.5%) compared to controls (4.4%) for age 18–64. This indicates not only severity differences by variant but also that severity differences differ by age with particularly low severity in age 18–64 Omicron cases.

### Post 3rd dose effectiveness by outcome

**Fig. 1:**
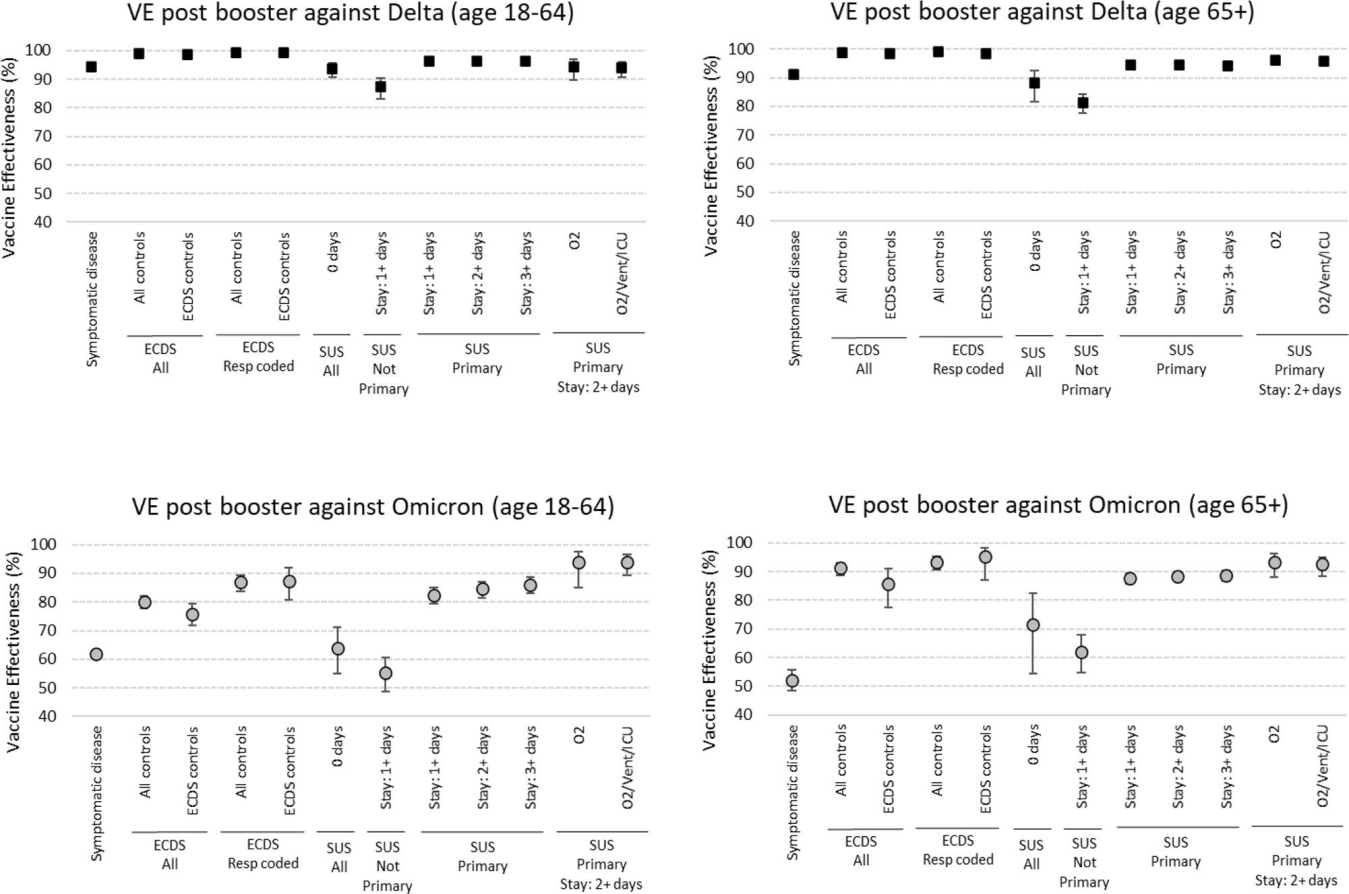
Vaccine effectiveness estimates with 95% confidence intervals 7+ days after a 3rd dose against symptomatic disease and different hospitalisation outcomes by age group and variant. ECDS Emergency Care Dataset—administrative data from emergency departments, SUS Secondary Users Service—administrative data from all secondary care which is coded on discharge, ECDS All all admissions through emergency care. ECDS Resp Coded respiratory SNOMED coded admission through emergency care, SUS All admitted with a respiratory ICD code, SUS Not Primary admitted with a respiratory ICD code not in the primary diagnosis field, SUS Primary admitted with a respiratory ICD code in the primary diagnosis field. 0 days, 1+ days, 2+ days, 3+ days: length of stay, for example, 1+ means at least one overnight stay. Vaccine effectiveness estimates are adjusted using sex, index of multiple deprivation (quintile), ethnic group, care home residence status (for age 65+), geographic region (NHS region), period (calendar week of test), health and social care worker status (for age <65), clinical risk group status (for age <65), clinically extremely vulnerable, severely immunosuppressed, and previously testing positive.

Figure 1 and Supplementary Table S3 summarise vaccine effectiveness at least 7 days post 3rd dose by age, variant and outcome. For Delta in those ages, 18–64 and 65+ VE against symptomatic infection was just over 90%. For all Delta ECDS analyses, VE was very high at over 98%, irrespective of controls used or respiratory coding or age. For the Delta SUS analysis, it is clear that those with 0 length of stay or not with a respiratory code in the primary field show lower VE, even lower than for symptomatic infection. The Delta SUS analyses with at least 2 days' stay and a primary coding all show VE of over 93%. For Omicron, results are much more variable. As previously seen, VE against symptomatic infection is much lower than for Delta with point estimates of 62% (age 18–64) and 52% (age 65+). For those age 65+, VE against hospitalisation using ECDS data is 86–91% improving to 93–95% if respiratory coding is used and with little variation according to which control group is used. VE in SUS in this age group is much lower, and more similar to symptomatic infection VE if using 0 days length of stay or a non-primary respiratory diagnosis. With the more specific and severe SUS endpoints VE increases to over 88–93% and is similar to that seen for ECDS. The picture in those aged 18–64 is more complex with ECDS data giving VE of 75–80% with an increase to 87% if respiratory coded. Using SUS data with 0 days of admission or a non-primary respiratory code gives VE similar to that for symptomatic infection, but there is a large increase in VE as the length of stay increases (to 89% VE) and with use of oxygen (to 93% VE). Only when oxygen use forms part of the definition is the VE in those aged 18–64 similar to that seen in age 65+.

### Effectiveness by vaccine manufacture, dose and interval

Analysis using all outcomes by dose intervals post vaccination are summarised in Table 1 (for Omicron) and Supplementary Table S4 (for Delta) with full details in Supplementary Tables S5–S10. They show the same general patterns as seen when concentrating on post 3rd dose effectiveness. With Delta, with almost all of the outcomes’, limited waning is seen, in particular among 18–64-year-olds. With Omicron waning is seen with the less specific and less severe outcomes, though this is less obvious with the more specific and more severe outcomes. More waning is seen among 18–64-year-olds with all outcomes for Omicron.

**Table 1:** Vaccine effectiveness against different hospitalisation outcomes with Omicron by dose and interval (all vaccines combined)

		Pillar 2s ECDS (ECDS controls)	Pillar 2s ECDS respiratory coded (ECDS respiratory controls)	Pillar 1/2 SUS All respiratory coded 0 days	Pillar 1/2 SUS not primary respiratory coded 1+ days	Pillar 1/2 SUS primary respiratory coded 1+ days	Pillar 1/2 SUS primary respiratory coded 2+ days	Pillar 1/2 SUS primary respiratory coded 3+ days	Pillar 1/2 SUS primary respiratory coded & oxygen 2+ days	Pillar 1/2 SUS primary respiratory coded & oxygen, ventilation, or ICU 2+ days
Age 18–64										
	Interval	VE (95% CI)	VE (95% CI)	VE (95% CI)	VE (95% CI)	VE (95% CI)	VE (95% CI)	VE (95% CI)	VE (95% CI)	VE (95% CI)
Dose 1	0–27	48.5 (12.3–69.7)	76.0 (−1.8 to 94.3)	21.9 (−89.7 to 67.9)		40.3 (−18 to 69.8)	36.2 (−33.9 to 69.6)	40 (−40.4 to 74.4)		
	28+	48.7 (32.8–60.8)	75.0 (50.3–87.4)	25.0 (−9.1 to 48.5)	16.2 (−3.7 to 32.3)	42.8 (26.3–55.5)	44.1 (25.6–58.0)	51.5 (33.2–64.8)	87.5 (55.6–96.5)	75.0 (42.4–89.1)
Dose 2	0–13	39.6 (−31.5 to 72.2)	50.0 (−187.1–91.3)	63.6 (−0.4 to 86.8)	8.3 (−152.9 to 66.7)	87.5 (59.5–96.1)	88.9 (58.4–97.0)	87.6 (53.7–96.7)		
	14–174	54.7 (45.3–62.4)	73.7 (56.9–84.0)	46.9 (30.5–59.5)	29.5 (15.1–41.5)	71.6 (63.4–77.9)	69.0 (58.1–77.0)	72.7 (61.4–80.7)	79.1 (−36.9 to 96.8)	86.7 (63.6–95.1)
	175+	34.6 (21.7–45.4)	46.5 (14.2–66.7)	41.7 (25.3–54.5)	17.8 (4.4–29.3)	52.5 (43.3–60.1)	56.1 (46.4–64.0)	60.6 (50.7–68.5)	80.5 (48.7–92.6)	82.3 (67.7–90.3)
3rd dose	0–6	63.9 (52.2–72.8)	76.3 (50.5–88.7)	62.6 (40.1–76.7)	19.8 (−25.2 to 48.6)	70.1 (51.8–81.4)	74.3 (55.9–85.0)	77.2 (59.5–87.2)	77.2 (−73.6 to 97.0)	90.7 (56.0–98.1)
	7–13	80.1 (73.5–85.1)	91.4 (82.7–95.7)	75.3 (61.1–84.3)	58.5 (39.3–71.6)	87.7 (79.9–92.5)	90.9 (83.2–95.1)	95.0 (89.1–97.7)		
	14–34	82.4 (78.6–85.6)	91.4 (85.5–94.9)	72.7 (63.9–79.3)	56.2 (47.3–63.7)	87.8 (84.3–90.5)	88.6 (84.9–91.5)	89.8 (85.9–92.6)	94.2 (76.6–98.6)	97.1 (92.2–98.9)
	35–69	72.7 (67.2–77.2)	86.2 (77.8–91.5)	62.6 (52–70.9)	56.6 (49.6–62.7)	83.4 (80.0–86.2)	85.8 (82.4–88.5)	87.8 (84.3–90.4)	93.9 (81.6–97.9)	94.3 (88.9–97.1)
	70–104	66.9 (59.1–73.3)	79.5 (64.5–88.1)	44.8 (26.1–58.8)	50.2 (40–58.7)	76.3 (70.8–80.7)	80.2 (74.9–84.4)	80.4 (74.5–85)	94.0 (78.8–98.3)	89.9 (78.3–95.3)
	105+	53.6 (36.9–65.9)	60.7 (14.7–81.9)	11.7 (−36.5 to 42.9)	50.9 (34.3–63.3)	66.3 (53.6–75.5)	67.4 (53.1–77.4)	68.6 (52.3–79.4)	80.4 (−36.3 to 97.2)	75.9 (15.8–93.1)
**Age 65**+										
	**Interval**	**VE (95% CI)**	**VE (95% CI)**	**VE (95% CI)**	**VE (95% CI)**	**VE (95% CI)**	**VE (95% CI)**	**VE (95% CI)**	**VE (95% CI)**	**VE (95% CI)**
Dose 1	0–27				11.7 (−118.6 to 64.3)	57.4 (−0.7 to 82.0)	43.9 (−41.0 to 77.7)	35.1 (−80.6 to 76.7)		
	28+			67.5 (6.7–88.6)	33.2 (5.2– 53.0)	52.3 (35.8–64.5)	53.4 (36.3–65.9)	57.6 (41.3–69.3)	48.3 (−169.1 to 90.0)	78.3 (43.7–91.7)
	14–174	77.8 (45.0–91.0)	90.6 (46.8–98.4)	55.1 (−131.8–91.3)	66.5 (47.1–78.7)	80.5 (72.2–86.3)	82.3 (74.3–87.8)	82.2 (73.7–87.9)	87.7 (50.9–96.9)	90.9 (72.6–97.0)
	175+	66.7 (43.4–80.4)	79.7 (34.4–93.7)	39.2 (−6.7 to 65.4)	23.5 (6.4– 37.5)	58.4 (51.0–64.7)	57.7 (49.6–64.4)	57.8 (49.4–64.9)	74.0 (47.6–87.1)	73.4 (55.1–84.3)
3rd dose	0–6	85.8 (61.5–94.7)	97.3 (79.2–99.7)	85.6 (6.4– 97.8)	38.9 (−2.6 to 63.6)	78.5 (66.8–86.1)	77.9 (65.3–85.9)	77.9 (64.7–86.2)	70.0 (−33.9 to 93.3)	89.2 (63.1–96.8)
	7–13	92.3 (76.3–97.5)	94.8 (−7.5 to 99.8)	69.2 (−18.7 to 92.0)	62.5 (40.7–76.4)	82.2 (73.0–88.3)	84.7 (76.0–90.2)	84.5 (75.5–90.2)	86.7 (−13.9 to 98.5)	94.7 (71.6–99.0)
	14–34	92.4 (86.0–95.8)	97.9 (92.6–99.4)	87.4 (72.5–94.2)	69.3 (60.8–76.0)	91.3 (89.1–93.0)	91.3 (89.1–93.1)	91.4 (89.0–93.2)	95.9 (89.0–98.4)	95.8 (91.3–97.9)
	35–69	87.0 (79.2–91.8)	95.3 (87.3–98.3)	79.3 (65.7–87.5)	67.2 (60.5–72.8)	88.9 (87.1–90.6)	89.3 (87.3–90.9)	89.5 (87.5–91.2)	93.9 (88.4–96.8)	92.8 (88.4–95.6)
	70–104	84.0 (74.6–89.9)	94.2 (84.0–97.9)	67.2 (46.6–79.8)	59.4 (51.5–66.0)	87.6 (85.6–89.3)	88.1 (86.1–89.9)	88.6 (86.5–90.3)	93.2 (87.5–96.2)	92.5 (88.1–95.2)
	105+	76.9 (60.6–86.4)	90.3 (67.8–97.1)	59.0 (30.5–75.8)	56.3 (46.9–64.0)	84.1 (81.2–86.5)	85.3 (82.4–87.6)	86.4 (83.6–88.7)	90.1 (79.7–95.2)	86.8 (77.1–92.3)

*ECDS* Emergency Care Dataset—administrative data from emergency departments, *SUS* Secondary Users Service—administrative data from all secondary care which is coded on discharge, *ECDS All* all admissions through emergency care, *ECDS Resp Coded* respiratory SNOMED coded admission through emergency care, *SUS All* admitted with a respiratory ICD code, *SUS Not Primary* admitted with a respiratory ICD code not in the primary diagnosis field, *SUS Primary* admitted with a respiratory ICD code in the primary diagnosis field.

**Fig. 2:**
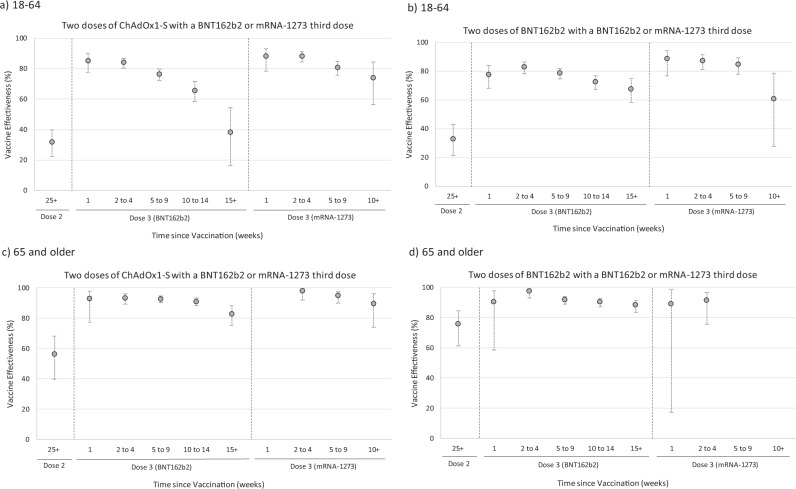
Vaccine effectiveness estimates with 95% confidence intervals against hospitalisations using ECDS by age group and manufacturer (all symptomatic controls, Omicron only). **a** 18–64: Two doses of ChAdOx1-S with a BNT162b2 or mRNA-1273 third dose, **b** 18–64: two doses of BNT162b2 with a BNT162b2 or mRNA-1273 third dose, **c** 65 and older: two doses of ChAdOx1-S with a BNT162b2 or mRNA-1273 third dose, **d** 65 and older: two doses of BNT162b2 with a BNT162b2 or mRNA-1273 third dose, Vaccine effectiveness estimates are adjusted using sex, index of multiple deprivation (quintile), ethnic group, care home residence status (for age 65+), geographic region (NHS region), period (calendar week of the test), health and social care worker status (for age <65), clinical risk group status (for age <65), clinically extremely vulnerable, severely immunosuppressed, and previously tested positive.

**Fig. 3:**
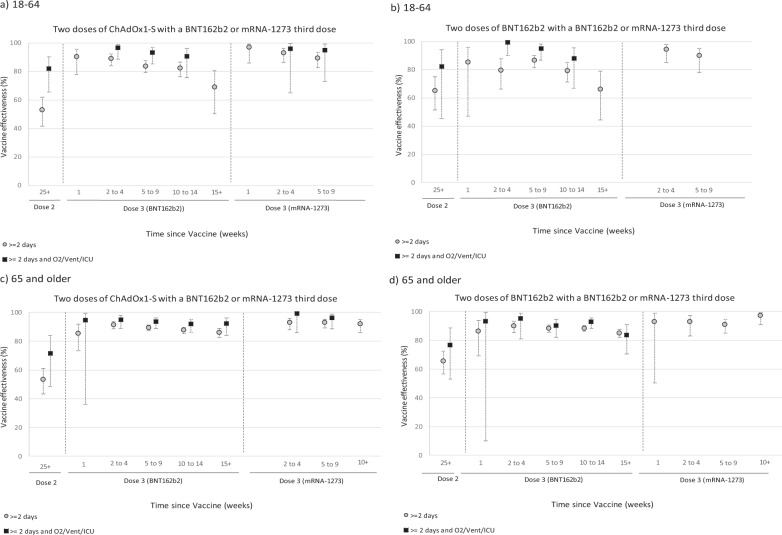
Vaccine effectiveness estimates with 95% confidence intervals against hospitalisations >=2 days and >=2 days and on oxygen/ventilated/on ICU using SUS by age group and manufacturer (all symptomatic controls, Omicron only). Vaccine effectiveness estimates are adjusted using sex, index of multiple deprivation (quintile), ethnic group, care home residence status (for age 65+), geographic region (NHS region), period (calendar week of the test), health and social care worker status (for age <65), clinical risk group status (for age <65), clinically extremely vulnerable, severely immunosuppressed, and previously tested positive. **a** 18–64: two doses of ChAdOx1-S with a BNT162b2 or mRNA-1273 third dose, **b** 18–64: two doses of BNT162b2 with a BNT162b2 or mRNA-1273 third dose, **c** 65 and older: two doses of ChAdOx1-S with a BNT162b2 or mRNA-1273 third dose, **d** 65 and older: two doses of BNT162b2 with a BNT162b2 or mRNA-1273 third dose.

To assess effectiveness by manufacturer, only the ECDS (all controls), ECDS respiratory coded (all controls), SUS primary code > =2 days stay and SUS, primary code > =2 days stay and Oxygen/ventilation/ICU endpoints were considered, and only for Omicron since Delta VE varies less by endpoint, and results have been previously published^16^. These endpoints were chosen to be the same ECDS endpoint used in past analyses and to use the more specific SUS endpoints. Figure 2 shows the ECDS analysis with lower VE in those aged 18–64 and waning post 3rd dose, more so for the BNT162b2 3rd dose where there is a longer follow-up where VE declines to 38% for those primed with ChAdOx1. For those aged 65+, ECDS VE is higher at over 90% up to 14 weeks post 3rd dose irrespective of priming vaccine or the 3rd dose received and remaining over 80% from 15+ weeks after the 3rd dose. In this age group, the waned 2 dose VE is higher for BNT162b2 (76%) than ChAdOx1 (56%). ECDS results with respiratory coding show generally higher VE with similar patterns (Supplementary Tables S5–S8). Figure 3 shows the SUS results and shows VE above 80% in almost all vaccination combinations and post 3rd dose periods. For those aged over 65+, the SUS data suggest little evidence of waning, whilst in those aged 18–64 VE declines to around 66–69% 15+ weeks after a BNT162b2 boost. Within each interval, VE is similar for both ChAdOx1 and BNT162b2 primed individuals and also for BNT162b2 and mRNA-1273-boosted individuals.

## Discussion

The results of this study demonstrate that assessment and interpretation of COVID-19 vaccine effectiveness against hospitalisation has become more complicated since the less severe Omicron variant has become dominant. When the disease is less severe a higher proportion of hospitalisations are likely to have COVID as an incidental finding rather than the cause of hospitalisation. This is the case for Omicron compared to Delta and for younger adults compared to older adults. Contamination of hospitalisations with these ‘incidental’ cases appears to result in lower vaccine effectiveness estimates against hospitalisation that are likely more reflective of vaccine effectiveness against infection. Vaccine effectiveness estimates improve and waning is more limited when definitions of hospitalisation that are more specific to severe respiratory disease are used.

For the Delta variant, we found that VE was fairly robust using the emergency care admissions or SUS-coded hospital discharges as long as the SUS respiratory discharge code was in the primary field and the admission length at least one overnight stay. The results suggest that for Delta a high proportion of these admissions are likely to be truly related to COVID-19 so that the VE measure is truly against a more severe disease. Furthermore, for Delta, contamination of the hospitalised cases with cases not hospitalised due to COVID will cause less bias for VE because symptomatic VE post 3rd dose is high. For the Omicron variant VE was also high and fairly robust to the case definition in those aged 65 and over, although it did increase when using respiratory coded ECDS admissions or when restricting to SUS cases with oxygen/ventilation or ICU. This age group also had the longest available follow-up post 3rd dose and largest numbers to look at VE by the specific vaccine with these results showing similar VE by schedule post 3rd dose and with VE remaining high to 15+ weeks after the 3rd dose. In those aged 18–64 VE was lower at below 90% unless additional interventions (oxygen, ventilation or ICU) were included. VE against Omicron was particularly low and similar to symptomatic disease VE if using those without a primary respiratory code or admitted and discharged on the same day. This suggests these cases may be asymptomatically identified cases from screening of all hospitalised patients. When assessing VE against Omicron, it is therefore not sufficient to just identify hospitalisation through routine hospital datasets without using more detailed data on diagnostic codes, length of stay and interventions. In previous reports, we have given VE against hospitalisation through ECDS (all ages) and this has suggested declines by time since 3rd dose^17^, but this current analysis indicates how this is likely to be due, at least in part, to many of these hospitalisations not being due to COVID-19 leading to the estimates mirroring the declines seen against symptomatic infection^8^. Using admissions of at least 2 days with a respiratory code in the primary diagnostic field VE in both age groups started at around 91% soon after the 3rd dose, dropping to around 67% by 15+ weeks in 18–64-year-olds and 85% in 65+ year-olds. Among those on oxygen VE went from around 94% down to 80% in 18–64-year-olds and 96% down to 90% in 65+ year-olds. The lower VE and more notable waning among 18–64-year-olds suggest that even with these more specific and more severe endpoints, there are likely to be a significant number of admissions where COVID is not the primary cause of their hospitalisation. Furthermore, among the 18–64 year-olds, those who first became eligible for vaccination, and thus have the longest follow-up, are those in clinical risk groups, including immunosuppressed individuals—this is likely to contribute to the greater apparent waning in the last follow-up period.

Our findings may go some way toward explaining the differing findings among existing studies of vaccine effectiveness against severe disease with the Omicron variant. For example, Abu-Raddad et al. found dose 3 vaccine effectiveness of 76.5% (95% CI, 55.9–87.5%) against Covid-19-related hospitalisation or death, which is lower than many other estimates^11^. This may be related to the fact that the study was dominated by under 60-year-olds, who in general, are likely to have milder disease. Other studies where VE estimates after three doses were over 90% have included older cohorts or have used physician manual review of medical notes to confirm the presence of severe COVID-19 symptoms^13,14^. We only identified two studies that had stratified by period after a 3rd dose—Thompson et al. found VE of 91% in the first 2 months following a third dose and 78% >= 4 months after the third dose—this is similar to our findings in 18–64-year-olds with some of the outcomes, though generally more waning than that we observed in 65+ year-olds^14^. In a study by Tartof et al.^18^ using emergency care and hospital admissions in the USA, also using a test-negative design, the effectiveness of BNT162b2 against hospital admission due to the omicron variant was 85% (95% CI 80–89) at less than 3 months but fell to 55% (28–71), at 3 months or longer the VE against emergency care admissions was lower at 77% (72–81) at less than 3 months falling to 53% (36–66) at 3 months or longer. This is comparable to our findings using the broadest definition of hospitalisation and likely represents an underestimate of VE against severe disease due to contamination with incidental admissions.

Our study also corresponds to finding from ref. 19 which used SpO2 levels and oxygen supplementation data to assess COVID-19 disease severity to measure the impact of vaccination on trends.

Test-negative case–control design has a number of limitations and advantages^8,16,20^. One of the biggest limitations of this specific study is that in relies on hospital-coded data which may have coding errors or not have interventions coded when they were used (e.g., oxygen use). A study where data are collected prospectively on cases using reporting forms or detailed case note review could avoid this misclassification bias, but is much more challenging to do with sufficiently large numbers^21^. One potential limitation for the TNCC design when looking at severe disease in controls is test sensitivity when a large proportion of those tested are truly positive. This, however, is more likely to affect Delta than Omicron analyses (as Delta is more severe) and is one of the reasons, along with study power, that in past analyses, we have chosen to use all symptomatic Pillar 2 controls for hospitalised COVID-19 VE. The analyses in this study do show slightly higher VE when using hospitalised Pillar 2 controls which may be due to this bias, but which may also be due to residual confounding from using all controls because it is necessary to adjust for factors related to risk of hospitalisation which is unlikely to be fully captured within the available adjustment covariates. Another limitation is that we have not done a formal validation on cases using more detailed case note review to show that those with short stays and coding not in primary fields are less likely to be admitted due to COVID-19. Examining differences in VE by vaccine is particularly challenging given differences in the populations that have received either vaccine. For example, those that received ChAdOx1-S as the primary course are more likely to be in clinical risk group, particularly among younger age groups. Similarly, those in the youngest age groups that were vaccinated earliest are likely to be in clinical risk groups. While adjustments are made for age and clinical risk group, there is likely to be residual confounding.

In conclusion, we found high levels of 3rd dose VE against hospitalisation with the Omicron variant, in particular among older adults who are at greatest risk, and against more severe endpoints. Nevertheless, there is evidence of limited waning from 3 to 4 months after a 3rd dose. Care should be taken in comparison of VE against hospitalisation across different studies due to the impact of using different outcome definitions.

## Methods

### Ethics

This research complies with all relevant ethical regulations as surveillance of coronavirus disease 2019 (Covid-19) testing and vaccination is undertaken under Regulation 3 of the Health Service (Control of Patient Information) Regulations 2002 to collect confidential patient information (www.legislation.gov.uk/uksi/2002/1438/regulation/3/made. opens in new tab) under Sections 3(i) (a) to (c), 3(i)(d) (i) and (ii), and 3. The study protocol was subject to an internal review by the Public Health England Research Ethics and Governance Group and was found to be fully compliant with all regulatory requirements ref:CAP-2021-07-UPDATE. Given that no regulatory issues were identified, and that ethics review is not a requirement for this type of work, it was decided that a full ethics review would not be necessary.

### Study design

A test-negative case–control design was used to estimate vaccine effectiveness in those aged 18 years and over against hospitalisation following a PCR test for SARS-CoV-2, as described previously^22–25^. Cases were those testing positive and controls those testing negative by PCR. Effectiveness was assessed using a variety of hospitalisation endpoints designed to differentiate between hospitalisations likely to be because of COVID-19 and those that may be hospitalisation with COVID-19 but potentially due to another cause. Effectiveness against Omicron and Delta was assessed using periods in which these variants were circulating and using the information on sequencing, genotyping and PCR s-gene target.

### Data sources

We linked COVID-19 PCR tests from both community Pillar 2 testing (wider population testing including drive/walk in and home testing) and in-hospital Pillar 1 testing (testing for those with a clinical need, health workers and travel) with vaccination data from the national vaccination register and hospitalisation data from either the Emergency Care Dataset (ECDS) or the Secondary Users Service (SUS). The SUS dataset is a national electronic database for all NHS hospital admissions in England with ICD-10 coded discharge coding for completed hospital stays. The ECDS includes hospital admissions through NHS emergency departments but not elective admissions with the reason for attending emergency care SNOMED coded.

The data sources are described in detail in Supplementary Text 1.

### Control selection

A maximum of one negative test per person within each of the following approximate 3-month periods was selected at random: 26 April to 1 August 2021, 2 August 2021 to 21 November 2021, 22 November 2021 to 23 February 2022. For analyses that involved hospitalised controls, any negative tests that led to hospitalisation within 21 days of a previous hospital negative test were excluded.

### Statistical analysis

Analysis was by logistic regression with the PCR test result as the dependent variable where those testing positives were cases and those testing negative controls. Vaccination status was included as an independent variable, and effectiveness defined as 1- odds of vaccination in cases/odds of vaccination in controls. Vaccination status was defined using date of onset, or, if missing or in Pillar 1 where this was not obtained, date of sample. Status was stratified by dose and interval post vaccination at 0–27 and 28+ days post first dose, 0–13, 14–174 and 175+ days post second dose and 0–6, 7–13,14–34, 35–69, 70–104, 105+ post 3rd dose. The analysis was also stratified by the manufacturer (ChAdOx1: Astrazeneca (adenoviral vector vaccine)or BNT162b2: Pfizer/BioNTech (mRNA vaccine) 2 dose priming, and BNT162b2: Pfizer/BioNTech (mRNA vaccine) or mRNA-1273: Moderna (mRNA vaccine)boosting) and by variant (Delta and Omicron). The analyses done to assess effectiveness according to the specificity of the hospitalisation are given in Table 2. The first analysis replicates those previously done for symptomatic infection and the following analyses using different criteria to allow comparison of emergency care and SUS data sources and to assess within SUS how VE changes based on whether the respiratory code is in the primary diagnostic field, the length of stay and the presence of codes for further interventions (oxygen, ventilator, ICU admission).

**Table 2:** analyses to assess vaccine effectiveness against symptomatic disease and hospitalisation endpoints

Analysis	Analysis name	Pillar of testing	Cases (test positive)	Controls (test negative)
1	Symptomatic disease	2	Symptomatic infection	Symptomatic infection (same as cases)
2	ECDS All—all controls	2	Symptomatic ECDS admitted	Symptomatic infection
3	ECDS All—ECDS controls	2	Symptomatic ECDS admitted	Symptomatic ECDS admitted (same as cases)
4	ECDS Respiratory coded—all controls	2	Symptomatic ECDS admitted with a Respiratory SNOMED code	Symptomatic infection
5	ECDS Respiratory coded—ECDS controls	2	Symptomatic ECDS admitted with a Respiratory SNOMED code	Symptomatic ECDS admitted with a Respiratory SNOMED code (same as cases)
6	SUS, 0 days	1 & 2	SUS admitted with a Respiratory ICD code with length of stay = 0	SUS admitted with a Respiratory ICD code with length of stay = 0(same as cases)
7	SUS, not primary, 1+ days	1 & 2	SUS admitted with length of stay ≥1 day and Respiratory ICD code not in the primary field	SUS admitted with length of stay ≥1 day and Respiratory ICD code not in the primary field (same as cases)
8–10	SUS, primary, stay 1+, 2+, 3+	1 & 2	SUS admitted, primary respiratory ICD code ≥1, ≥2, ≥3 days stay	SUS admitted, primary respiratory ICD code ≥1, ≥2, ≥3 days stay (same as cases)
11	SUS, primary, stay 2+, O2	1 & 2	SUS admitted, primary respiratory ICD code, ≥2 days stay, oxygen use	SUS admitted, primary respiratory ICD code, ≥2 days stay, oxygen use (same as cases)
12	SUS, primary, stay 2+, O2/Vent/ICU	1 & 2	SUS admitted, Respiratory ICD code primary, ≥2 days stay, oxygen use or ventilation of ICU admission.	SUS admitted, Respiratory ICD code primary, ≥2 days stay, oxygen use or ventilation of ICU admission. (same as cases)

*ECDS* Emergency Care Dataset—administrative data from emergency departments, *SUS* Secondary Users Service—administrative data from all secondary care which is coded on discharge.

Pillar 1 testing: clinical need, health workers and travel.

Pillar 2 testing: wider population testing, including drive/walk in and home testing.

Vaccine effectiveness was adjusted in logistic regression models for age (5-year bands), sex, index of multiple deprivation (quintile), ethnic group, care home residence status (for age 65+), geographic region (NHS region), period (calendar week of test), health and social care worker status (for age <65), clinical risk group status (for age <65), clinically extremely vulnerable, severely immunosuppressed, and previously testing positive. The logistic model for the log-odds of the probability of being a positive case therefore included all of these explanatory variables as well as the variable for vaccination status. All analyses were stratified by age 18–64 and 65+. For the vaccine manufacturer stratification, only endpoints 2–5, 9 and 12 were considered and only for Omicron. Numbers were too small in those primed with mRNA-1273 to assess this schedule (Supplementary Table S1).

All analyses were conducted in STATA 17™.

### Reporting summary

Further information on research design is available in the Nature Research Reporting Summary linked to this article.

## Supplementary information

Supplementary Information

Supplementary Table 17

Reporting Summary

## Data Availability

The raw vaccine effectiveness data are protected and are not available due to data privacy laws. This work is carried out under Regulation 3 of The Health Service (Control of Patient Information) (Secretary of State for Health, 2002)^3^ using patient identification information without individual patient consent. Data cannot be made publicly available for ethical and legal reasons, i.e., public availability would compromise patient confidentiality as data tables list single counts of individuals rather than aggregated data.

## Code availability

Available upon request.
