# Supplementary material for: Effectiveness of COVID-19 vaccines against Omicron and Delta hospitalisation, a test negative case-control study
Source: Nat Commun. 2022 Sep 30;13:5736. doi: 10.1038/s41467-022-33378-7 (PMC9523190; doi:10.1038/s41467-022-33378-7)
Supplement: Supplementary file 1 — Supplementary Information [file 41467_2022_33378_MOESM1_ESM.pdf]

Supplementary Text S1: Data sources

COVID-19 Testing Data

PCR testing for SARS CoV-2 in England is undertaken by hospital and public health laboratories (Pillar 1), as well as by community testing (Pillar 2). Pillar 2 testing was available to anyone with symptoms consistent with COVID-19 (high temperature, new continuous cough, or loss or change in sense of smell or taste), anyone who was a contact of a confirmed case, care home staff and residents, and to those who tested positive using a lateral flow test (LFT). Pillar 1 testing is PCR testing in public health laboratories and NHS hospitals and was available for inpatients and others presenting to secondary care as well as health and care workers.

Data on all positive tests regardless of symptom status (PCR and LFT) and negative PCR tests from Pillar 2 from symptomatic individuals and all Pillar 1 tests with a sample date from 25 November 2020 to 10 March 2022 were identified. Where participants had a positive test within 14 days of another positive, the earliest PCR test was used, but sequencing, genotyping and S-gene target status from later tests was retained. Where tests were on the same day pillar 2 symptomatic tests were retained. Positive and negative tests within 90 days of a previous positive test were excluded and negative tests taken within 21 days after a positive test were excluded. Data were restricted to tests with a valid NHS number so linkage to the vaccination record could be carried out.

Classification of positive samples as Delta and Omicron variants was done using, in order of priority, whole genome sequencing, genotyping and S-gene target status. From sequencing Omicron is VOC-21NOV-01 or VUI-22JAN-01and Delta is VOC-21APR-02 or VUI-21OCT-01. S-gene target failure as well as genotyping and sequencing data could be used to identify Omicron from 29 November 2021 onward, however from 10 January 2022 Delta was very rare so all samples were assigned as Omicron if no sequencing or genotyping was done.(1) The Delta variant was classified using only sequencing, genotyping and S-gene target non-failure for the periods of 26 April 2021- 23 May 2021 and 22 November 2021 to 3 January 2022, whilst for the period from 24 May to 21 November 2022, all samples were defined as Delta if no sequencing, genotyping or S-gene testing was done. Only those individuals where the variant was classified as Delta or Omicron were retained for analysis. The study period was therefore 26 April 2021 to 3 January 2022 for Delta and 22 November 2022 to2 February 2022 for Omicron.

Vaccination Data

These testing data were linked to the vaccination histories and demographic characteristics of the populations on 14 March 2022 using The National Immunisation Management System (NIMS) as previously described.(1, 2) Third doses were identified as a 3rd dose given at least 84 days after a second dose and administered after 13 September 2021. Individuals with vaccination histories outside the recommended schedules were excluded from the analysis (Figure S1).

Testing data were linked to NIMS on 14 March 2022 using combinations of the unique individual National Health Service (NHS) number, date of birth, surname, first name, and postcode using deterministic linkage.

Emergency Care Hospital Admission Data

Emergency Care hospital admissions from the Emergency Care Dataset (ECDS), which includes hospital admissions through emergency departments but not elective admissions, were linked using NHS number and date of birth to the testing data on 15 March 2022 to identify admissions within 14 days of a community test. Admissions due to an injury were excluded. Admissions were identified where the Emergency Care Destination code was either discharge to a ward, intensive care unit, coronary care unit, high dependency unit or where there was a date on which the decision to admit the patient was made. Admissions with the reason for attending emergency care being a SNOMED CT (Systematized Nomenclature of Medicine–Clinical Terms) coded acute respiratory illness were flagged.(3)

Secondary Care Hospital Admission Data

Hospital inpatient admissions for a range of acute respiratory illnesses were identified from the Secondary Uses Service (SUS). SUS is the national electronic database of hospital admissions that provides timely updates of ICD-10 codes for completed hospital stays for all NHS hospitals in England. Up to 24 ICD-10 diagnoses fields can be completed in SUS for each admission with the first diagnosis field indicating the primary reason for admission. Oxygen use and ventilation support was ascertained using the Classification of Interventions and Procedures (OPCS-4) codes (table S15). Intensive Care Unit (ICU) admission status was ascertained by the Main Specialty of the ward being Critical Care Medicine or the Treatment Function being Intensive Care Medicine. Length of stay was calculated as date of discharge – date of admission.

For the Pillar 2 samples admissions with an ICD-10 acute respiratory illness discharge diagnosis, in any diagnosis field, were identified where the sample was taken 14 days before and up to 2 days after the day of admission. For the Pillar 1 samples, admissions with an ICD-10 coded respiratory discharge diagnosis in any diagnosis field, were identified where the sample was taken 1 days before and up to 2 days after the admission. The data was restricted to tests up to 23 February 2022 to account for delays in the SUS data recording. Linkage to the testing data was carried out on 15 March 2022 using NHS number and date of birth. Where multiple admissions linked to the same sample date the first admission after the sample date was retained and episode length calculated by summing the stay length for each admission.

Table S1. Descriptive characteristics of positive and negative test results in individuals 18-64 years of age tested for SARS-CoV-2 in England for the study population

			Delta				Omicron			
			**Positive**		**Negative**		**Positive**		**Negative**	
			**n**	**%**	**n**	**%**	**n**	**%**	**n**	**%**
**Category**			**52,155**		**81,821**		**19,790**		**22,548**	
**Vaccination Status after vaccine**	**Manufacturer (primary course)**	**Dose**								
		Unvaccinated	29,877	57.3%	13,942	17.0%	5,136	26.0%	2,822	12.5%
	ChAdOx1-S	Dose 1	1,825	3.5%	6,180	7.6%	409	2.1%	447	2.0%
		Dose 2	13,385	25.7%	31027	37.9%	2401	12.1%	3930	17.4%
		3rd dose (any)	368	0.7%	5210	6.4%	4578	23.1%	7310	32.4%
	BNT162b2	Dose 1	2672	5.1%	4014	4.9%	761	3.8%	581	2.6%
		Dose 2	3548	6.8%	16820	20.6%	2473	12.5%	2807	12.4%
		3rd dose (any)	235	0.5%	3637	4.4%	3708	18.7%	4280	19.0%
	mRNA-1273	Dose 1	187	0.4%	387	0.5%	66	0.3%	73	0.3%
		Dose 2	58	0.1%	572	0.7%	154	0.8%	217	1.0%
		3rd dose (any)	0	0.0%	32	0.0%	104	0.5%	81	0.4%
**Outcomes**	ECDS admissions -pillar 2 symptomatic		17,232	100.0%	19,461	100.0%	3,519	100.0%	6,432	100.0%
	ECDS admissions -pillar 2 symptomatic with respiratory code		9,618	55.8%	3,070	15.8%	782	22.2%	973	15.1%
	Secondary Uses Service Respiratory coded admissions (SUS)		47,550	100.0%	63,781	100.0%	17,075	100.0%	16,527	100.0%
	Secondary Uses Service Respiratory coded admissions (SUS) with 0 days length of stay		5,766	12.1%	7,808	12.2%	5,626	32.9%	2,333	14.1%
	SUS with respiratory code but NOT in primary diagnosis field with at least 1 day stay		9,395	19.8%	29,601	46.4%	7,469	43.7%	7,308	44.2%
	SUS with respiratory code as the primary diagnosis and length of stay >=1 day		32,389	68.1%	26,372	41.3%	3,980	23.3%	6,886	41.7%
	SUS with respiratory code as the primary diagnosis and length of stay >=2 days		28,537	60.0%	21,834	34.2%	2,827	16.6%	5,568	33.7%
	SUS with respiratory code as the primary diagnosis and length of stay >=3 days		25,163	52.9%	18,193	28.5%	2,110	12.4%	4,606	27.9%
	SUS >=2 days stay and primary diagnosis with oxygen use		5,866	12.3%	1,385	2.2%	222	1.3%	336	2.0%
	SUS >=2 days stay and primary diagnosis with oxygen use or ventilation used or in ICU		9,898	20.8%	2,980	4.7%	424	2.5%	729	4.4%

Testing route	Pillar 1	24,007	46.0%	58,249	71.2%	10,457	52.8%	15,055	66.8%
	Pillar 2	28,148	54.0%	23,572	28.8%	9,333	47.2%	7,493	33.2%
Gender	Female	27,731	53.2%	43,406	53.0%	13,042	65.9%	12,064	53.5%
	Male	24,338	46.7%	37,772	46.2%	6,717	33.9%	10,317	45.8%
	Missing	86	0.2%	643	0.8%	31	0.2%	167	0.7%
Age	18-19	1,024	2.0%	1,893	2.3%	545	2.8%	509	2.3%
	20-24	3,507	6.7%	4,690	5.7%	1838	9.3%	1219	5.4%
	25-29	5,288	10.1%	5,515	6.7%	2586	13.1%	1,588	7.0%
	30-34	6,479	12.4%	6,607	8.1%	2872	14.5%	1,908	8.5%
	35-39	6,276	12.0%	6,683	8.2%	2263	11.4%	1,960	8.7%
	40-44	5,797	11.1%	6,789	8.3%	2000	10.1%	1,805	8.0%
	45-49	5,611	10.8%	7,980	9.8%	1714	8.7%	2,172	9.6%
	50-54	6,153	11.8%	11,059	13.5%	1955	9.9%	3,024	13.4%
	55-59	6,281	12.0%	14,123	17.3%	2035	10.3%	3,833	17.0%
	60-64	5,739	11.0%	16,482	20.1%	1982	10.0%	4,530	20.1%
Ethnicity	Missing	3,714	7.1%	3,938	4.8%	1,074	5.4%	1,182	5.2%
	African	1,597	3.1%	1,529	1.9%	606	3.1%	378	1.7%
	Any other Asian background	1136	2.2%	1,240	1.5%	397	2.0%	324	1.4%
	Any other Black background	869	1.7%	735	0.9%	224	1.1%	175	0.8%
	Any other White background	5,296	10.2%	5,160	6.3%	1,845	9.3%	1,407	6.2%
	Any other ethnic group	1,669	3.2%	1,460	1.8%	608	3.1%	415	1.8%
	Any other mixed background	501	1.0%	467	0.6%	173	0.9%	113	0.5%
	Bangladeshi or British Bangladeshi	731	1.4%	636	0.8%	250	1.3%	147	0.7%
	British, Mixed British	30,215	57.9%	60,081	73.4%	12455	62.9%	16661	73.9%
	Caribbean	1,356	2.6%	857	1.0%	289	1.5%	206	0.9%
	Chinese	245	0.5%	184	0.2%	60	0.3%	71	0.3%
	Indian or British Indian	1,522	2.9%	2,245	2.7%	646	3.3%	616	2.7%
	Irish	208	0.4%	590	0.7%	117	0.6%	157	0.7%
	Pakistani or British Pakistani	2,360	4.5%	2,006	2.5%	786	4.0%	528	2.3%
	White and Asian	181	0.3%	184	0.2%	69	0.3%	45	0.2%
	White and Black African	191	0.4%	188	0.2%	66	0.3%	55	0.2%
	White and Black Caribbean	364	0.7%	321	0.4%	125	0.6%	68	0.3%
NHS Region	East of England	4,507	8.6%	7,886	9.6%	1,799	9.1%	2,067	9.2%
	London	8,343	16.0%	10,895	13.3%	3,314	16.7%	2,703	12.0%
	Midlands	11,370	21.8%	17,289	21.1%	4,160	21.0%	5,060	22.4%
	North East	9,393	18.0%	13,400	16.4%	3,260	16.5%	3,813	16.9%
	North West	8,423	16.1%	13,772	16.8%	3,104	15.7%	3,669	16.3%
	South East	5,463	10.5%	11,085	13.5%	2609	13.2%	3,146	14.0%
	South West	4,656	8.9%	7,494	9.2%	1544	7.8%	2,090	9.3%
IMD Quintiles	1	16,559	31.7%	24,343	29.8%	6,103	30.8%	6,675	29.6%
	2	12,173	23.3%	18,626	22.8%	4,503	22.8%	5,061	22.4%
	3	9,193	17.6%	14,926	18.2%	3,600	18.2%	4,182	18.5%
	4	7,621	14.6%	12,983	15.9%	2,930	14.8%	3,697	16.4%
	5	6,259	12.0%	10,408	12.7%	2,552	12.9%	2,836	12.6%
	Missing	350	0.7%	535	0.7%	102	0.5%	97	0.4%
**Previously positive**	No	51,292	98.3%	71,560	87.5%	17,583	88.8%	18,748	83.1%
	Yes	863	1.7%	10,261	12.5%	2,207	11.2%	3,800	16.9%

**Vaccine priority groups**	Healthcare worker	1,354	2.6%	3,216	3.9%	1,098	5.5%	939	4.2%
	CEV	8,311	15.9%	24,526	30.0%	5,123	25.9%	6,245	27.7%
	At risk	22,997	44.1%	51,771	63.3%	9,967	50.4%	13,907	61.7%
	Severely immunosuppressed	1,458	2.8%	4,939	6.0%	1,550	7.8%	1,352	6.0%

Table S2: Descriptive characteristics of positive and negative test results in individuals 65 years of age and over tested for SARS-CoV-2 in England for the study population

			Delta				Omicron			
			Positive		Negative		Positive		Negative	
			n	%	n	%	n	%	n	%
Category			30,427		187,463		13,348		49,643	
Vaccination Status after vaccine	Manufacturer (primary course)	Dose								
		Unvaccinated	5,033	16.5%	6676	3.3%	1452	1.1%	1462	3%
	ChAdOx1-S	Dose 1	590	1.9%	5,399	2.7%	153	9.7%	331	1%
		Dose 2	14073	46.3%	70923	35.0%	1295	35.1%	4057	8%
		3rd dose (any)	921	3.0%	19693	9.7%	4679	1.0%	20015	40%
	BNT162b2	Dose 1	336	1.1%	2237	1.1%	133	4.5%	256	1%
		Dose 2	8270	27.2%	58131	28.7%	607	37.6%	2275	5%
		3rd dose (any)	1197	3.9%	24358	12.0%	5016	0.1%	21227	43%
	mRNA-1273	Dose 1	6	0.0%	30	0.0%	11	0.0%	11	0%
		Dose 2	1	0.0%	15	0.0%	2	0.0%	8	0%
		3rd dose (any)	0	0.0%	1	0.0%	0	8.8%	1	0%
Outcomes	ECDS admissions -pillar 2 symptomatic		5,331	100.0%	6,059	100.0%	1174	100.0%	2,317	100.0%
	ECDS admissions -pillar 2 symptomatic with respiratory code		3,170	59.5%	1,559	25.7%	467	39.8%	547	23.6%
	Secondary Uses Service Respiratory coded admissions (SUS)		29,596	100.0%	183,108	100.0%	12,250	100.0%	47,931	100.0%
	Secondary Uses Service Respiratory coded admissions (SUS) with 0 days length of stay		989	3.3%	6,450	3.5%	1,393	11.4%	2,121	4.4%
	SUS with respiratory code but NOT in primary diagnosis field with at least 1 day stay		5,446	18.4%	82,963	45.3%	4,704	38.4%	20,067	41.9%
	SUS with respiratory code as the primary diagnosis and length of stay >=1 day		23,161	78.3%	93,695	51.2%	6,153	50.2%	25,743	53.7%
	SUS with respiratory code as the primary diagnosis and length of stay >=2 days		21,808	73.7%	85,806	46.9%	5,565	45.4%	23,257	48.5%
	SUS with respiratory code as the primary diagnosis and length of stay >=3 days		20,271	68.5%	77,998	42.6%	5,019	41.0%	20,796	43.4%
	SUS >=2 days stay and primary diagnosis with oxygen use		3,625	12.2%	4,092	2.2%	517	4.2%	1,155	2.4%
	SUS >=2 days stay and primary diagnosis with oxygen use or ventilation used or in ICU		6,265	21.2%	7,146	3.9%	807	6.6%	1,921	4.0%
Testing route	Pillar 1		20,659	67.9%	179,756	95.9%	9,482	71.0%	46,858	94.4%
	Pillar 2		9,768	32.1%	7,707	4.1%	3,866	29.0%	2,785	5.6%

Gender	Female	13,739	45.2%	94,863	50.6%	6,379	47.8%	25,660	51.7%
	Male	16,668	54.8%	91,485	48.8%	6,947	52.0%	23,756	47.9%
	Missing	20	0.1%	1,115	0.6%	22	0.2%	227	0.5%
Age	65-69	5,515	18.1%	19,569	10.4%	1,932	14.5%	5,375	10.8%
	70-74	6,456	21.2%	28,250	15.1%	2,368	17.7%	7,556	15.2%
	75-79	6,021	19.8%	33,227	17.7%	2,540	19.0%	8,899	17.9%
	80-84	5,296	17.4%	36,800	19.6%	2,534	19.0%	9,906	20.0%
	85-89	4,255	14.0%	36,298	19.4%	2,251	16.9%	9,571	19.3%
	>=90	2,884	9.5%	33,319	17.8%	1,723	12.9%	8,336	16.8%
Ethnicity	Missing	1,412	4.6%	7,838	4.2%	579	4.3%	2,221	4.5%
	African	238	0.8%	597	0.3%	105	0.8%	131	0.3%
	Any other Asian background	308	1.0%	1,165	0.6%	134	1.0%	294	0.6%
	Any other Black background	152	0.5%	298	0.2%	50	0.4%	79	0.2%
	Any other White background	1,559	5.1%	8,230	4.4%	684	5.1%	2,163	4.4%
	Any other ethnic group	354	1.2%	1,297	0.7%	145	1.1%	314	0.6%
	Any other mixed background	79	0.3%	469	0.3%	41	0.3%	102	0.2%
	Bangladeshi or British Bangladeshi	207	0.7%	518	0.3%	68	0.5%	114	0.2%
	British, Mixed British	23,386	76.9%	158,305	84.4%	10,445	78.3%	42,004	84.6%
	Caribbean	645	2.1%	1,179	0.6%	248	1.9%	273	0.5%
	Chinese	92	0.3%	244	0.1%	32	0.2%	57	0.1%
	Indian or British Indian	797	2.6%	2,941	1.6%	292	2.2%	798	1.6%
	Irish	317	1.0%	2,370	1.3%	186	1.4%	585	1.2%
	Pakistani or British Pakistani	740	2.4%	1,671	0.9%	296	2.2%	428	0.9%
	White and Asian	37	0.1%	109	0.1%	9	0.1%	26	0.1%
	White and Black African	24	0.1%	79	0.0%	5	0.0%	20	0.0%
	White and Black Caribbean	80	0.3%	153	0.1%	29	0.2%	34	0.1%
-NHS Region	East of England	2,666	8.8%	20,728	11.1%	1,227	9.2%	5,013	10.1%
	London	3,670	12.1%	20,657	11.0%	1,952	14.6%	4,858	9.8%
	Midlands	6,224	20.5%	37,399	20.0%	2,643	19.8%	11,046	22.3%
	North East	6,832	22.5%	29,448	15.7%	2,501	18.7%	8,136	16.4%
	North West	4,838	15.9%	28,624	15.3%	2,180	16.3%	7,386	14.9%
	South East	3,305	10.9%	30,103	16.1%	1,859	13.9%	7,909	15.9%
	South West	2,892	9.5%	20,504	10.9%	986	7.4%	5,295	10.7%
IMD Quintiles	1	8,146	26.8%	37,552	20.0%	3,379	25.3%	9,855	19.9%
	2	6,581	21.6%	37,515	20.0%	2,726	20.4%	9,933	20.0%
	3	5,734	18.8%	38,002	20.3%	2,626	19.7%	10,023	20.2%
	4	5,457	17.9%	38,607	20.6%	2,423	18.2%	10,333	20.8%
	5	4,441	14.6%	35,434	18.9%	2,165	16.2%	9,420	19.0%
	Missing	68	0.2%	353	0.2%	29	0.2%	79	0.2%
**Previously positive**	No	29,963	41.6%	171,099	23.9%	12,649	94.8%	44,761	90.2%
	Yes	464	2.3%	16,364	2.6%	699	5.2%	4,882	9.8%
**Vaccine priority groups**	Healthcare worker	63	0.2%	262	0.1%	30	0.2%	74	0.1%
	CEV	13,802	45.4%	98,148	52.4%	7,054	52.8%	24,200	48.7%
	Severely immunosuppressed	1,893	6.2%	11,918	6.4%	1,434	10.7%	3,598	7.2%

Table S3: Vaccine effectiveness (and 95% CI) at least 7 days after the third dose of COVID-19 vaccine using ECDS and secondary care hospital admission data by the Delta and Omicron variants by age group

**Age 18-64**	**Omicron**			**Delta**		
**Outcome**	**cases**	**controls**	**VE ≥7 days post 3rd dose**	**cases**	**controls**	**VE ≥7 days post 3rd dose**
**P2s all cases and controls**	713,260	827,973	61.9 (61.5 to 62.3)	18,686	560,146	94.2 (94.1 to 94.3)
**P2s ECDS (all controls)**	1,472	827,973	80.0 (77.8 to 82.0)	115	560,146	99.1 (98.9 to 99.3)
**P2s ECDS (ECDS controls)**	1,472	3,268	75.9 (71.7 to 79.5)	115	2,167	98.7 (98.4 to 99.0)
**P2s ECDS respiratory coded (all controls)**	323	827,973	86.9 (83.8 to 89.4)	54	560,146	99.4 (99.2 to 99.5)
**P2s ECDS respiratory coded (ECDS respiratory controls)**	323	479	87.4 (80.8 to 91.8)	54	361	99.3 (99.0 to 99.5)
**P1/2 SUS respiratory coded 0 days**	3,291	1,061	63.9 (55.0 to 71.1)	36	740	93.6 (90.6 to 95.7)
**P1/2 SUS 1+ days Not Primary**	2,201	3,408	55.2 (48.9 to 60.7)	63	2,425	87.3 (83.0 to 90.5)
**P1/2 SUS 1+ days Primary**	1,436	3,462	82.3 (79.3 to 84.9)	192	2,658	96.4 (95.6 to 97.0)
**P1/2 SUS 2+ days Primary**	921	2,834	84.5 (81.5 to 87.1)	173	2,209	96.3 (95.5 to 97.0)
**P1/2 SUS 3+ days Primary**	640	2,337	86.1 (83.0 to 88.6)	154	1,859	96.4 (95.5 to 97.1)
**P1/2 SUS 2+ days Primary & oxygen**	46	168	93.9 (85.0 to 97.5)	42	133	94.4 (89.8 to 96.9)
**P1/2 SUS 2+ days Primary & oxygen, ventilation, or ICU**	87	368	94.0 (89.3 to 96.6)	67	297	93.9 (90.7 to 96.1)
**Age 65+**	**Omicron**			**Delta**		
**Outcome**	**cases**	**controls**	**VE ≥7 days post 3rd dose**	**cases**	**controls**	**VE ≥7 days post 3rd dose**
**P2s all cases and controls**	102,720	137,812	52.3 (48.3 to 55.9)	7,384	130,830	91.1 (90.6 to 91.6)
**P2s ECDS (all controls)**	908	137,812	91.3 (88.9 to 93.1)	203	130,830	98.8 (98.6 to 99)
**P2s ECDS (ECDS controls)**	908	2,034	85.7 (77.5 to 90.9)	203	1,941	98.4 (97.8 to 98.8)
**P2s ECDS respiratory coded (all controls)**	358	137,812	93.4 (90.6 to 95.3)	105	130,830	99.1 (98.8 to 99.3)
**P2s ECDS respiratory coded (ECDS respiratory controls)**	358	481	95.2 (87.0 to 98.2)	105	521	98.6 (97.7 to 99.2)
**P1/2 SUS 0 days**	1,203	1,778	71.6 (54.4 to 82.3)	50	1,568	88.4 (81.6 to 92.6)
**P1/2 SUS 1+ days Not Primary**	3,595	16,111	62.0 (54.8 to 68)	318	15,966	81.4 (77.7 to 84.4)
**P1/2 SUS 1+ days Primary**	3,934	20,822	87.7 (85.9 to 89.3)	981	21,215	94.5 (93.9 to 95.0)
**P1/2 SUS 2+ days Primary**	3,501	18,765	88.3 (86.4 to 89.9)	913	19,259	94.5 (93.8 to 95.0)
**P1/2 SUS 3+ days Primary**	3,109	16,702	88.7 (86.8 to 90.4)	847	17,266	94.3 (93.6 to 94.9)

**P1/2 SUS 2+ days Primary & oxygen**	287	913	93.3 (88.2 to 96.2)	145	997	96.2 (94.6 to 97.4)
**P1/2 SUS 2+ days Primary & oxygen, ventilation, or ICU**	451	1,516	92.5 (88.5 to 95.1)	247	1,642	95.9 (94.7 to 96.9)

P2s: Pillar 2 symptomatic *P1/2: Pillar 1 and 2*

ECDS: Emergency Care Dataset -administrative data from emergency departments. SUS: Secondary Users Service -administrative data from all secondary care which is coded on discharge.

ECDS All: All admissions through emergency care; ECDS Resp Coded: Respiratory SNOMED coded admission through emergency care; SUS All: Admitted with a respiratory ICD code; SUS Not Primary: Admitted with a respiratory ICD code not in the primary diagnosis field; SUS Primary: Admitted with a respiratory ICD code in the primary diagnosis field.
0 days, 1+ days, 2+ days, 3+ days: Length of stay, for example 1+ means at least one overnight stay

Vaccine effectiveness estimates are adjusted using sex, index of multiple deprivation (quintile), ethnic group, care home residence status (for age 65+), geographic region (NHS region), period (calendar week of test), health and social care worker status (for age <65), clinical risk group status (for age<65), clinically extremely vulnerable, severely immunosuppressed, and previously testing positive

Table S4: Vaccine effectiveness (95% CI) against different hospitalisation outcomes with Delta by dose and interval (all vaccines combined)

		P2s ECDS (ECDS controls)	P2s ECDS respiratory coded (ECDS respiratory controls)	P1/2 SUS 0 days	P1/2 SUS 1+ days Not Primary	P1/2 SUS 1+ days Primary	P1/2 SUS 2+ days Primary	P1/2 SUS 3+ days Primary	P1/2 SUS 2+ days Primary & oxygen	P1/2 SUS 2+ days Primary & oxygen, ventilation, or ICU
	**18-64**									
Dose 1	0-27	67.1 (61.8 to 71.6)	69.6 (58.4 to 77.7)	35.8 (14.8 to 51.6)	40.3 (24.8 to 52.6)	44.7 (34.3 to 53.5)	43.5 (31.1 to 53.6)	46.3 (32.8 to 57.1)	41.4 (-14.8 to 70.1)	51.0 (18.7 to 70.4)
	28+	85 (83.4 to 86.5)	89.2 (86.6 to 91.4)	65.1 (59.0 to 70.3)	54.7 (49.5 to 59.3)	82.7 (81.0 to 84.3)	82.7 (80.8 to 84.4)	83.0 (80.9 to 84.8)	83.3 (76.7 to 88.0)	82.7 (78.0 to 86.4)
Dose 2	0-13	92.8 (91.3 to 94.1)	94.6 (92.0 to 96.4)	83.4 (76.9 to 88.1)	63.2 (51.6 to 72.0)	90.5 (88.1 to 92.5)	90.8 (88.0 to 92.9)	91.1 (88.2 to 93.3)	80.0 (55.7 to 91.0)	85.0 (73.0 to 91.7)
	14-174	93.1 (92.5 to 93.6)	95.0 (94.1 to 95.8)	82.5 (80.3 to 84.4)	71.1 (68.7 to 73.2)	91.0 (90.4 to 91.5)	91.1 (90.5 to 91.7)	91.0 (90.3 to 91.7)	91.4 (89.2 to 93.1)	90.5 (88.9 to 91.9)
	175+	90.4 (89.2 to 91.5)	93.3 (91.5 to 94.7)	75.0 (69.7 to 79.3)	61.8 (56.1 to 66.7)	84.2 (82.5 to 85.7)	84.5 (82.7 to 86.1)	85.2 (83.3 to 86.8)	86.1 (80.3 to 90.2)	84.3 (79.9 to 87.7)
3rd dose	0-6	94.7 (93.3 to 95.9)	97.0 (95.4 to 98.0)	86.7 (78.2 to 91.8)	76.7 (64.6 to 84.7)	88.9 (85.7 to 91.5)	90.0 (86.7 to 92.5)	90.3 (86.8 to 92.9)	94.0 (83.9 to 97.8)	92.7 (86.1 to 96.1)
	7-13	98.5 (97.7 to 99.0)	99.3 (98.6 to 99.6)	92.5 (83.9 to 96.5)	86.4 (76.9 to 92.1)	95.0 (93.1 to 96.4)	94.8 (92.6 to 96.3)	94.7 (92.4 to 96.4)	95.0 (81.4 to 98.6)	93.9 (87.2 to 97.1)
	14-34	99.0 (98.6 to 99.3)	99.5 (99.1 to 99.7)	95.3 (91.4 to 97.4)	87.3 (80.9 to 91.6)	97.5 (96.7 to 98.2)	97.4 (96.4 to 98.1)	97.6 (96.6 to 98.3)	96.0 (90.4 to 98.3)	95.2 (90.7 to 97.5)
	35-69	98.5 (97.9 to 99.0)	99.0 (98.1 to 99.4)	94.0 (88.0 to 97.0)	87.4 (77.4 to 93.0)	96.0 (94.3 to 97.2)	96.1 (94.3 to 97.3)	96.0 (94.1 to 97.4)	93.2 (83.4 to 97.3)	93.5 (86.2 to 97.0)
	70-104	98.7 (95.9 to 99.6)	99.1 (95.5 to 99.8)	71.8 (21.2 to 89.9)		89.7 (75.9 to 95.6)	91.3 (75.4 to 96.9)	91.7 (76.0 to 97.1)		
	**65+**									
Dose 1	0-27					55.1 (35.7 to 68.7)	59.2 (41.1 to 71.8)	55.0 (33.8 to 69.4)		

	28+	84.5 (76.1 to 90)	89.8 (79.1 to 95)	65.4 (37.0 to 81.0)	40.3 (27.4 to 50.9)	74.7 (71.6 to 77.6)	74.5 (71.2 to 77.4)	74.2 (70.7 to 77.3)	82.3 (71.7 to 89.0)	86.7 (81.4 to 90.5)
Dose 2	0-13	85.1 (60.7 to 94.3)	81.4 (26.7 to 95.3)	66.8 (-64.5 to 93.3)	48.3 (6.8 to 71.4)	82.8 (75.0 to 88.2)	81.8 (73.3 to 87.6)	82.7 (74.0 to 88.5)	88.1 (64.4 to 96.0)	89.8 (74.5 to 95.9)
	14-174	89.6 (86.6 to 91.9)	89.1 (83.0 to 93.0)	76.0 (67.0 to 82.6)	57.7 (52.4 to 62.4)	85.7 (84.7 to 86.6)	85.6 (84.6 to 86.6)	85.7 (84.7 to 86.7)	90.1 (87.3 to 92.2)	90.5 (88.6 to 92.1)
	175+	85.0 (80.5 to 88.5)	85.1 (76.3 to 90.6)	56.6 (38.8 to 69.2)	46.7 (39.3 to 53.1)	78.2 (76.6 to 79.8)	78.3 (76.6 to 79.9)	78.1 (76.3 to 79.7)	83.9 (78.8 to 87.8)	84.9 (81.5 to 87.7)
3rd dose	0-6	91.7 (88.3 to 94.1)	91.9 (85.3 to 95.5)	81.8 (66.9 to 90.0)	54.4 (43.1 to 63.5)	86.8 (84.8 to 88.5)	86.9 (84.8 to 88.6)	86.4 (84.2 to 88.3)	93.5 (89.2 to 96.0)	92.1 (88.4 to 94.6)
	7-13	97.3 (96.0 to 98.2)	97.6 (95.3 to 98.8)	77.8 (58.6 to 88.1)	71.3 (63.2 to 77.7)	88 (86.2 to 89.6)	88.6 (86.8 to 90.1)	88.3 (86.4 to 90.0)	91.6 (86.1 to 94.9)	92.3 (88.9 to 94.7)
	14-34	98.9 (98.4 to 99.3)	99.0 (98.2 to 99.4)	90.8 (83.7 to 94.8)	83.9 (80.0 to 87.1)	96.4 (95.8 to 96.9)	96.3 (95.7 to 96.8)	96.1 (95.5 to 96.6)	97.7 (96.3 to 98.5)	97.5 (96.5 to 98.3)
	35-69	98.4 (97.7 to 98.9)	98.7 (97.5 to 99.3)	90.9 (81.1 to 95.6)	85.4 (80.5 to 89.1)	95.7 (94.9 to 96.4)	95.7 (94.8 to 96.4)	95.6 (94.7 to 96.3)	96.7 (94.4 to 98.1)	96.1 (94.0 to 97.4)
	70-104	97.7 (95.5 to 98.8)	98.0 (94.6 to 99.3)		55.7 (-23.9 to 84.2)	91.2 (85.2 to 94.8)	90.2 (83.5 to 94.3)	89.7 (82.1 to 94.1)	90.0 (43.4 to 98.2)	87.0 (58.6 to 95.9)

P2s: Pillar 2 symptomatic *P1/2: Pillar 1 and 2*

ECDS: Emergency Care Dataset -administrative data from emergency departments. SUS: Secondary Users Service -administrative data from all secondary care which is coded on discharge.

ECDS All: All admissions through emergency care; ECDS Resp Coded: Respiratory SNOMED coded admission through emergency care; SUS All: Admitted with a respiratory ICD code; SUS Not Primary: Admitted with a respiratory ICD code not in the primary diagnosis field; SUS Primary: Admitted with a respiratory ICD code in the primary diagnosis field.
0 days, 1+ days, 2+ days, 3+ days: Length of stay, for example 1+ means at least one overnight stay

Vaccine effectiveness estimates are adjusted using sex, index of multiple deprivation (quintile), ethnic group, care home residence status (for age 65+), geographic region (NHS region), period (calendar week of test), health and social care worker status (for age <65), clinical risk group status (for age<65), clinically extremely vulnerable, severely immunosuppressed, and previously testing positive

Table S5: Vaccine effectiveness against hospital admissions from emergency care (ECDS) within 14 days of the test date by the Delta and Omicron variants in symptomatic individuals 18 to 64 years of age

**Omicron**							
		**Pillar 2 symptomatic with ECDS admission within 14 days**			**Pillar 2 symptomatic with respiratory coded ECDS admission within 14 days**		
	**Interval (days)**	**cases**	**controls**	**VE (95% CI)**	**cases**	**controls**	**VE (95% CI)**
**unvaccinated**		**653**	**497**		**177**	**66**	
Dose 1	0-27	28	43	48.5 (12.3 to 69.7)	6	4	
	28+	148	226	48.7 (32.8 to 60.8)	28	39	75.0 (50.3 to 87.4)
Dose 2	0-13	13	24	39.6 (-31.5 to 72.2)	3	5	
	14-174	441	1,111	54.7 (45.3 to 62.4)	75	188	73.7 (56.9 to 84.0)
	175+	659	968	34.6 (21.7 to 45.4)	152	142	46.5 (14.2 to 66.7)
3rd dose	0-6	105	295	63.9 (52.2 to 72.8)	18	50	76.3 (50.5 to 88.7)
	7-13	88	328	80.1 (73.5 to 85.1)	17	67	91.4 (82.7 to 95.7)
	14-34	288	1,013	82.4 (78.6 to 85.6)	52	148	91.4 (85.5 to 94.9)
	35-69	584	1,248	72.7 (67.2 to 77.2)	116	178	86.2 (77.8 to 91.5)
	70-104	377	538	66.9 (59.1 to 73.3)	100	69	79.5 (64.5 to 88.1)
	105+	135	141	53.6 (36.9 to 65.9)	38	17	60.7 (14.7 to 81.9)
**Delta**							
	**Interval (days)**	**cases**	**controls**	**VE (95% CI)**	**cases**	**controls**	**VE (95% CI)**
**unvaccinated**		**9,170**	**2,696**		**5,447**	**381**	
Dose 1	0-27	486	574	67.1 (61.8 to 71.6)	262	81	69.6 (58.4 to 77.7)
	28+	999	1,868	85.0 (83.4 to 86.5)	456	279	89.2 (86.6 to 91.4)
Dose 2	0-13	161	551	92.8 (91.3 to 94.1)	76	78	94.6 (92.0 to 96.4)
	14-174	4,982	9,434	93.1 (92.5 to 93.6)	2,611	1,511	95.0 (94.1 to 95.8)
	175+	1,192	1,786	90.4 (89.2 to 91.5)	643	304	93.3 (91.5 to 94.7)
3rd dose	0-6	127	385	94.7 (93.3 to 95.9)	69	75	97.0 (95.4 to 98.0)
	7-13	32	394	98.5 (97.7 to 99.0)	13	85	99.3 (98.6 to 99.6)
	14-34	43	897	99.0 (98.6 to 99.3)	19	144	99.5 (99.1 to 99.7)
	35-69	37	694	98.5 (97.9 to 99.0)	20	104	99.0 (98.1 to 99.4)
	70-104	3	179	98.7 (95.9 to 99.6)	2	26	99.1 (95.5 to 99.8)
	105+	0	3		0	2	

Vaccine effectiveness estimates are adjusted using sex, index of multiple deprivation (quintile), ethnic group, care home residence status (for age 65+), geographic region (NHS region), period (calendar week of test), health and social care worker status (for age <65), clinical risk group status (for age<65), clinically extremely vulnerable, severely immunosuppressed, and previously testing positive

Table S6: Vaccine effectiveness against hospital admissions from emergency care (ECDS) within 14 days of the test date by the Delta and Omicron variants in symptomatic individuals 18 to 64 years of age using all controls

**Omicron**							
		**Pillar 2 symptomatic with ECDS admission within 14 days**			**Pillar 2 symptomatic with respiratory coded ECDS admission within 14 days**		
	**Interval (days)**	**cases**	**controls**	**VE (95% CI)**	**cases**	**controls**	**VE (95% CI)**
**unvaccinated**		**653**	**127,778**		**177**	**127,778**	
Dose 1	0-27	28	9832	41.1 (13.8 to 59.7)	6	9,832	50.6 (-11.9 to 78.2)
	28+	148	48,886	39.2 (27.0 to 49.3)	28	48,886	58.6 (37.6 to 72.5)
Dose 2	0-13	13	7354	56.9 (25.2 to 75.2)	3	7,354	60.9 (-22.7 to 87.6)
	14-174	441	440,896	59.3 (53.9 to 64.1)	75	440,896	71.7 (62.5 to 78.6)
	175+	659	212,640	33.4 (25.3 to 40.6)	152	212,640	50.3 (37.5 to 60.5)
3rd dose	0-6	105	100,217	67.8 (60.2 to 73.9)	18	100,217	80.1 (67.3 to 87.9)
	7-13	88	106,474	83.3 (79.0 to 86.7)	17	106,474	88.9 (81.4 to 93.4)
	14-34	288	281,633	85.5 (83.3 to 87.5)	52	281,633	91.1 (87.7 to 93.6)
	35-69	584	306,848	79.1 (76.3 to 81.5)	116	306,848	87.7 (84.1 to 90.5)
	70-104	377	106,966	70.6 (66.0 to 74.6)	100	106,966	79.5 (72.8 to 84.6)
	105+	135	26,052	60.5 (51.2 to 68.1)	38	26,052	72.5 (58.6 to 81.8)
**Delta**							
	**Interval (days)**	**cases**	**controls**	**VE (95% CI)**	**cases**	**controls**	**VE (95% CI)**
**unvaccinated**		**9170**	**566,636**		**5447**	**566,636**	
Dose 1	0-27	486	159,118	74.1 (71.6 to 76.5)	262	159,118	75.3 (71.9 to 78.3)
	28+	999	391,467	86.9 (86.0 to 87.8)	456	391,467	89.9 (88.9 to 90.9)
Dose 2	0-13	161	122309	94.2 (93.2 to 95.0)	76	122,309	95.6 (94.4 to 96.5)
	14-174	4982	2,218,985	94.7 (94.5 to 94.9)	2611	2,218,985	95.7 (95.4 to 95.9)
	175+	1192	363,447	92.0 (91.4 to 92.5)	643	363,447	93.1 (92.5 to 93.7)
3rd dose	0-6	127	117,633	95.9 (95.1 to 96.6)	69	117,633	96.5 (95.6 to 97.3)
	7-13	32	117,498	98.9 (98.5 to 99.2)	13	117,498	99.3 (98.8 to 99.6)
	14-34	43	236,845	99.3 (99.1 to 99.5)	19	236,845	99.5 (99.2 to 99.7)
	35-69	37	157,782	99.0 (98.6 to 99.3)	20	157,782	99.1 (98.7 to 99.5)
	70-104	3	47,688	99.3 (97.8 to 99.8)	2	47,688	99.2 (97.0 to 99.8)
	105+	0	333		0	333	

Vaccine effectiveness estimates are adjusted using sex, index of multiple deprivation (quintile), ethnic group, care home residence status (for age 65+), geographic region (NHS region), period (calendar week of test), health and social care worker status (for age <65), clinical risk group status (for age<65), clinically extremely vulnerable, severely immunosuppressed, and previously testing positive

Table S7: Vaccine effectiveness against hospital admissions from emergency care (ECDS) within 14 days of the test date by the Delta and Omicron variants in symptomatic individuals 65 years of age and older

**Omicron**							
		**Pillar 2 symptomatic with ECDS admission within 14 days**			**Pillar 2 symptomatic with respiratory coded ECDS admission within 14 days**		
	**Interval (days)**	**cases**	**controls**	**VE (95% CI)**	**cases**	**controls**	**VE (95% CI)**
**unvaccinated**		**104**	**39**		**49**	**10**	
Dose 1	0-27	4	6		3	0	
	28+	17	13		6	2	
Dose 2	14-174	13	25	77.8 (45.0 to 91.0)	5	8	90.6 (46.8 to 98.4)
	175+	119	160	66.7 (43.4 to 80.4)	44	36	79.7 (34.4 to 93.7)
3rd dose	0-6	9	40	85.8 (61.5 to 94.7)	2	10	97.3 (79.2 to 99.7)
	7-13	6	56	92.3 (76.3 to 97.5)	2	10	94.8 (-7.5 to 99.8)
	14-34	31	300	92.4 (86.0 to 95.8)	10	86	97.9 (92.6 to 99.4)
	35-69	263	888	87.0 (79.2 to 91.8)	101	218	95.3 (87.3 to 98.3)
	70-104	423	633	84.0 (74.6 to 89.9)	166	137	94.2 (84.0 to 97.9)
	105+	185	157	76.9 (60.6 to 86.4)	79	30	90.3 (67.8 to 97.1)
**Delta**							
	**Interval (days)**	**cases**	**controls**	**VE (95% CI)**	**cases**	**controls**	**VE (95% CI)**
**unvaccinated**		**701**	**115**		**458**	**32**	
Dose 1	0-27	15	8	59.2 (-12.0 to 85.1)	8	0	
	28+	78	130	84.5 (76.1 to 90.0)	40	37	89.8 (79.1 to 95.0)
Dose 2	0-13	7	82	85.1 (60.7 to 94.3)	5	17	81.4 (26.7 to 95.3)
	14-174	2,742	2,831	89.6 (86.6 to 91.9)	1,634	690	89.1 (83.0 to 93.0)
	175+	1,417	780	85.0 (80.5 to 88.5)	826	216	85.1 (76.3 to 90.6)
3rd dose	0-6	168	172	91.7 (88.3 to 94.1)	94	46	91.9 (85.3 to 95.5)
	7-13	57	172	97.3 (96.0 to 98.2)	29	43	97.6 (95.3 to 98.8)
	14-34	63	583	98.9 (98.4 to 99.3)	38	171	99.0 (98.2 to 99.4)
	35-69	68	864	98.4 (97.7 to 98.9)	31	224	98.7 (97.5 to 99.3)
	70-104	15	322	97.7 (95.5 to 98.8)	7	83	98.0 (94.6 to 99.3)

Vaccine effectiveness estimates are adjusted using sex, index of multiple deprivation (quintile), ethnic group, care home residence status (for age 65+), geographic region (NHS region), period (calendar week of test), health and social care worker status (for age <65), clinical risk group status (for age<65), clinically extremely vulnerable, severely immunosuppressed, and previously testing positive

Table S8: Vaccine effectiveness against hospital admissions from emergency care (ECDS) within 14 days of the test date by the Delta and Omicron variants in symptomatic individuals 18 to 64 years of age using all controls

		Pillar 2 symptomatic with ECDS admission within 14 days			Pillar 2 symptomatic with respiratory coded ECDS admission within 14 days		
	**Interval (days)**	**cases**	**controls**	**VE (95% CI)**	**cases**	**controls**	**VE (95% CI)**
**unvaccinated**		**104**	**1,703**		**49**	**1,703**	
Dose 1	0-27	4	211	58.4 (-23.1 to 85.9)	3	211	16.7 (-182.9 to 75.5)
	28+	17	640	62.9 (35.5 to 78.7)	6	640	73.8 (36.0 to 89.2)
Dose 2	0-13	0	30		0	30	
	14-174	13	977	80.8 (64.1 to 89.7)	5	977	86.7 (64.6 to 95.0)
	175+	119	6,250	63.4 (50.5 to 72.9)	44	6,250	76.6 (63.0 to 85.2)
3rd dose	0-6	9	2,117	81.6 (62.0 to 91.1)	2	2,117	93.1 (70.4 to 98.4)
	7-13	6	3,810	93.6 (85.0 to 97.2)	2	3,810	96.0 (83.0 to 99.0)
	14-34	31	25,524	95.1 (92.5 to 96.8)	10	25,524	97.0 (93.9 to 98.5)
	35-69	263	67,132	92.7 (90.5 to 94.4)	101	67,132	94.6 (92.0 to 96.3)
	70-104	423	34,337	90.6 (87.9 to 92.7)	166	34,337	92.9 (89.7 to 95.1)
	105+	185	7,009	86.2 (81.2 to 89.8)	79	7,009	88.6 (82.1 to 92.7)
**Delta**							
	**Interval (days)**	**cases**	**controls**	**VE (95% CI)**	**cases**	**controls**	**VE (95% CI)**
**unvaccinated**		**701**	**4,552**		**458**	**4,552**	
Dose 1	0-27	15	487	67.2 (43.4 to 80.9)	8	487	70.8 (39.7 to 85.8)
	28+	78	4,460	83.3 (78.5 to 87.0)	40	4,460	86.8 (81.4 to 90.6)
Dose 2	0-13	7	3164	88.9 (76.1 to 94.9)	5	3164	86.7 (66.9 to 94.7)
	14-174	2,742	143,293	90.8 (89.8 to 91.7)	1,634	143,293	91.5 (90.4 to 92.5)
	175+	1,417	38,227	87.6 (86.1 to 88.9)	826	38,227	89.1 (87.4 to 90.5)
3rd dose	0-6	168	9,216	93.2 (91.7 to 94.3)	94	9,216	94.3 (92.7 to 95.5)
	7-13	57	10,883	97.9 (97.3 to 98.5)	29	10,883	98.4 (97.6 to 98.9)
	14-34	63	38,663	99.2 (98.9 to 99.4)	38	38,663	99.2 (98.9 to 99.5)
	35-69	68	63,758	99.0 (98.6 to 99.2)	31	63,758	99.3 (98.9 to 99.5)
	70-104	15	17,461	98.2 (96.8 to 99.0)	7	17,461	98.7 (97.1 to 99.4)
	105+	0	65		0	65	

Vaccine effectiveness estimates are adjusted using sex, index of multiple deprivation (quintile), ethnic group, care home residence status (for age 65+), geographic region (NHS region), period (calendar week of test), health and social care worker status (for age <65), clinical risk group status (for age<65), clinically extremely vulnerable, severely immunosuppressed, and previously testing positive

Table S9: Vaccine effectiveness using secondary care hospital admission data (SUS) by the Delta and Omicron variants in individuals 18-64 years of age

		Omicron			Delta		
		**SUS Pillar1 and 2 (0 days stay in hospital)**					
**Interval (days)**		**cases**	**controls**	**VE (95% CI)**	**cases**	**controls**	**VE (95% CI)**
**unvaccinated**		**914**	**321**		**3,021**	**1,444**	
Dose 1	0-27	24	11	21.9 (-89.7 to 67.9)	197	149	35.8 (14.8 to 51.6)
	28+	216	94	25.0 (-9.1 to 48.5)	486	756	65.1 (59.0 to 70.3)
Dose 2	0-13	18	11	63.6 (-0.4 to 86.8)	71	222	83.4 (76.9 to 88.1)
	14-174	443	318	46.9 (30.5 to 59.5)	1,604	3,519	82.5 (80.3 to 84.4)
	175+	645	434	41.7 (25.3 to 54.5)	326	843	75.0 (69.7 to 79.3)
3rd dose	0-6	75	83	62.6 (40.1 to 76.7)	25	135	86.7 (78.2 to 91.8)
	7-13	81	90	75.3 (61.1 to 84.3)	8	125	92.5 (83.9 to 96.5)
	14-34	368	301	72.7 (63.9 to 79.3)	13	306	95.3 (91.4 to 97.4)
	35-69	949	434	62.6 (52.0 to 70.9)	10	240	94.0 (88.0 to 97.0)
	70-104	1,197	194	44.8 (26.1 to 58.8)	5	66	71.8 (21.2 to 89.9)
	105+	696	42	11.7 (-36.5 to 42.9)	0	3	
		**SUS Pillar1 and 2 respiratory code not in primary diagnostic field but 1+ days stay**					
		**cases**	**controls**	**VE (95% CI)**	**cases**	**controls**	**VE (95% CI)**
**unvaccinated**		**2,472**	**1,133**		**5,032**	**5,625**	
Dose 1	0-27	64	32	0.0 (-92.6 to 48.0)	200	424	40.3 (24.8 to 52.6)
	28+	530	339	16.2 (-3.7 to 32.3)	876	3,634	54.7 (49.5 to 59.3)
Dose 2	0-13	26	14	8.3 (-152.9 to 66.7)	102	778	63.2 (51.6 to 72.0)
	14-174	841	666	29.5 (15.1 to 41.5)	2602	13,340	71.1 (68.7 to 73.2)
	175+	1,252	1,536	17.8 (4.4 to 29.3)	486	3,032	61.8 (56.1 to 66.7)
3rd dose	0-6	83	180	19.8 (-25.2 to 48.6)	34	343	76.7 (64.6 to 84.7)
	7-13	88	279	58.5 (39.3 to 71.6)	17	431	86.4 (76.9 to 92.1)
	14-34	512	884	56.2 (47.3 to 63.7)	30	980	87.3 (80.9 to 91.6)
	35-69	960	1,464	56.6 (49.6 to 62.7)	16	844	87.4 (77.4 to 93.0)
	70-104	490	619	50.2 (40.0 to 58.7)	0	168	
	105+	151	162	50.9 (34.3 to 63.3)	0	2	
		**SUS Pillar1 and 2 at least 1 day stay and respiratory code in primary diagnostic field**					
		**cases**	**controls**	**VE (95% CI)**	**cases**	**controls**	**VE (95% CI)**
**unvaccinated**		**1,307**	**865**		**20,009**	**4,235**	
Dose 1	0-27	29	33	40.3 (-18 to 69.8)	755	356	44.7 (34.3 to 53.5)
	28+	237	337	42.8 (26.3 to 55.5)	1560	2,896	82.7 (81.0 to 84.3)
Dose 2	0-13	7	19	87.5 (59.5 to 96.1)	192	725	90.5 (88.1 to 92.5)
	14-174	214	596	71.6 (63.4 to 77.9)	7571	12,237	91.0 (90.4 to 91.5)
	175+	705	1,385	52.5 (43.3 to 60.1)	1946	2,905	84.2 (82.5 to 85.7)
3rd dose	0-6	45	189	70.1 (51.8 to 81.4)	164	360	88.9 (85.7 to 91.5)
	7-13	40	313	87.7 (79.9 to 92.5)	65	484	95.0 (93.1 to 96.4)
	14-34	194	921	87.8 (84.3 to 90.5)	66	1,079	97.5 (96.7 to 98.2)
	35-69	504	1,389	83.4 (80 to 86.2)	53	903	96.0 (94.3 to 97.2)
	70-104	509	690	76.3 (70.8 to 80.7)	8	191	89.7 (75.9 to 95.6)
	105+	189	149	66.3 (53.6 to 75.5)	0	1	

		SUS Pillar1 and 2 at least 2 days stay and respiratory code in primary diagnostic field					
		**cases**	**controls**	**VE (95% CI)**	**cases**	**controls**	**VE (95% CI)**
**unvaccinated**		**1,016**	**696**		**17836,**	**3,473**	
Dose 1	0-27	25	24	36.2 (-33.9 to 69.6)	633	270	43.5 (31.1 to 53.6)
	28+	178	272	44.1 (25.6 to 58.0)	1,326	2,410	82.7 (80.8 to 84.4)
Dose 2	0-13	5	15	88.9 (58.4 to 97.0)	157	605	90.8 (88.0 to 92.9)
	14-174	135	430	69.0 (58.1 to 77.0)	6,543	10,137	91.1 (90.5 to 91.7)
	175+	517	1,150	56.1 (46.4 to 64.0)	1,732	2,438	84.5 (82.7 to 86.1)
3rd dose	0-6	30	147	74.3 (55.9 to 85.0)	137	292	90.0 (86.7 to 92.5)
	7-13	23	252	90.9 (83.2 to 95.1)	58	394	94.8 (92.6 to 96.3)
	14-34	134	762	88.6 (84.9 to 91.5)	63	908	97.4 (96.4 to 98.1)
	35-69	330	1,133	85.8 (82.4 to 88.5)	47	745	96.1 (94.3 to 97.3)
	70-104	310	567	80.2 (74.9 to 84.4)	5	161	91.3 (75.4 to 96.9)
	105+	124	120	67.4 (53.1 to 77.4)	0	1	
		**SUS Pillar1 and 2 at least 3 days stay and respiratory code in primary diagnostic field**					
		**cases**	**controls**	**VE (95% CI)**	**cases**	**controls**	**VE (95% CI)**
**unvaccinated**		**804**	**560**		**15819**	**2,863**	
Dose 1	0-27	20	19	40.0 (-40.4 to 74.4)	525	213	46.3 (32.8 to 57.1)
	28+	133	234	51.5 (33.2 to 64.8)	1129	2,039	83.0 (80.9 to 84.8)
Dose 2	0-13	5	12	87.6 (53.7 to 96.7)	134	500	91.1 (88.2 to 93.3)
	14-174	91	338	72.7 (61.4 to 80.7)	5738	8,405	91.0 (90.3 to 91.7)
	175+	391	984	60.6 (50.7 to 68.5)	1541	2,074	85.2 (83.3 to 86.8)
3rd dose	0-6	26	122	77.2 (59.5 to 87.2)	123	240	90.3 (86.8 to 92.9)
	7-13	13	212	95.0 (89.1 to 97.7)	52	334	94.7 (92.4 to 96.4)
	14-34	96	637	89.8 (85.9 to 92.6)	55	766	97.6 (96.6 to 98.3)
	35-69	230	937	87.8 (84.3 to 90.4)	42	621	96.0 (94.1 to 97.4)
	70-104	222	463	80.4 (74.5 to 85.0)	5	137	91.7 (76.0 to 97.1)
	105+	79	88	68.6 (52.3 to 79.4)	0	1	
		**SUS Pillar1 and 2 at least 2 days stay & oxygen with respiratory code in primary diagnostic field**					
		**cases**	**controls**	**VE (95% CI)**	**cases**	**controls**	**VE (95% CI)**
**unvaccinated**		**126**	**42**		**3,918**	**258**	
Dose 1	0-27	1	1		119	19	41.4 (-14.8 to 70.1)
	28+	9	18	87.5 (55.6 to 96.5)	221	154	83.3 (76.7 to 88.0)
Dose 2	0-13	0	1		39	30	80.0 (55.7 to 91.0)
	14-174	6	18	79.1 (-36.9 to 96.8)	1184	599	91.4 (89.2 to 93.1)
	175+	30	81	80.5 (48.7 to 92.6)	322	175	86.1 (80.3 to 90.2)
3rd dose	0-6	4	7		21	17	94.0 (83.9 to 97.8)
	7-13	0	13		8	18	95.0 (81.4 to 98.6)
	14-34	6	43	94.2 (76.6 to 98.6)	13	50	96.0 (90.4 to 98.3)
	35-69	15	75	93.9 (81.6 to 97.9)	18	55	93.2 (83.4 to 97.3)
	70-104	19	30	94 (78.8 to 98.3)	3	10	
	105+	6	7	80.4 (-36.3 to 97.2)			
		**SUS Pillar1 and 2 at least 2 days stay & either oxygen, ventilation or ICU with respiratory code in primary diagnostic field**					
		**cases**	**controls**	**VE (95% CI)**	**cases**	**controls**	**VE (95% CI)**

**unvaccinated**		**227**	**87**		**6,511**	**505**	
Dose 1	0-27	5	3		174	34	51.0 (18.7 to 70.4)
	28+	22	32	75.0 (42.4 to 89.1)	376	331	82.7 (78.0 to 86.4)
Dose 2	0-13	0	3		52	73	85.0 (73.0 to 91.7)
	14-174	12	47	86.7 (63.6 to 95.1)	2,090	1,338	90.5 (88.9 to 91.9)
	175+	67	172	82.3 (67.7 to 90.3)	583	360	84.3 (79.9 to 87.7)
3rd dose	0-6	4	17	90.7 (56 to 98.1)	45	42	92.7 (86.1 to 96.1)
	7-13	0	28		18	51	93.9 (87.2 to 97.1)
	14-34	9	107	97.1 (92.2 to 98.9)	23	118	95.2 (90.7 to 97.5)
	35-69	31	155	94.3 (88.9 to 97.1)	22	107	93.5 (86.2 to 97.0)
	70-104	36	66	89.9 (78.3 to 95.3)	4	21	
	105+	11	12	75.9 (15.8 to 93.1)			

Vaccine effectiveness estimates are adjusted using sex, index of multiple deprivation (quintile), ethnic group, care home residence status (for age 65+), geographic region (NHS region), period (calendar week of test), health and social care worker status (for age <65), clinical risk group status (for age<65), clinically extremely vulnerable, severely immunosuppressed, and previously testing positive

Table S10: Vaccine effectiveness using secondary care hospital admission data (SUS) by the Delta and Omicron variants in individuals 65 years of age and older

		Omicron			Delta		
		SUS Pillar1 and 2 (0 days stay in hospital)					
	**Interval (days)**	**cases**	**controls**	**VE (95% CI)**	**cases**	**controls**	**VE (95% CI)**
**unvaccinated**		**69**	**60**		**104**	**202**	
Dose 1	0-27	1	6		4	17	
	28+	14	25	67.5 (6.7 to 88.6)	20	214	65.4 (37.0 to 81.0)
Dose 2	0-13				2	95	66.8 (-64.5 to 93.3)
	14-174	6	13	55.1 (-131.8 to 91.3)	521	3,240	76.0 (67.0 to 82.6)
	175+	97	196	39.2 (-6.7 to 65.4)	266	949	56.6 (38.8 to 69.2)
3rd dose	0-6	3	43	85.6 (6.4 to 97.8)	22	165	81.8 (66.9 to 90.0)
	7-13	5	36	69.2 (-18.7 to 92)	18	135	77.8 (58.6 to 88.1)
	14-34	25	192	87.4 (72.5 to 94.2)	21	481	90.8 (83.7 to 94.8)
	35-69	211	741	79.3 (65.7 to 87.5)	11	704	90.9 (81.1 to 95.6)
	70-104	551	565	67.2 (46.6 to 79.8)	0	245	
	105+	411	244	59.0 (30.5 to 75.8)	0	3	
		**SUS Pillar1 and 2 respiratory code not in primary diagnostic field but 1+ days stay**					
	**Interval (days)**	**cases**	**controls**	**VE (95% CI)**	**cases**	**controls**	**VE (95% CI)**
**unvaccinated**		**354**	**636**		**479**	**3,194**	
Dose 1	0-27	12	17		21	161	
	28+	82	228	33.2 (5.2 to 53.0)	174	3,450	40.3 (27.4 to 50.9)
Dose 2	0-13	0	15		13	1,339	48.3 (6.8 to 71.4)
	14-174	35	273	66.5 (47.1 to 78.7)	2,765	43,778	57.7 (52.4 to 62.4)
	175+	595	2,399	23.5 (6.4 to 37.5)	1,535	13,500	46.7 (39.3 to 53.1)
3rd dose	0-6	31	388	38.9 (-2.6 to 63.6)	141	1,575	54.4 (43.1 to 63.5)
	7-13	33	496	62.5 (40.7 to 76.4)	105	1,686	71.3 (63.2 to 77.7)
	14-34	179	2,267	69.3 (60.8 to 76.0)	140	5,188	83.9 (80.0 to 87.1)
	35-69	805	6,378	67.2 (60.5 to 72.8)	68	6,759	85.4 (80.5 to 89.1)
	70-104	1,718	5,148	59.4 (51.5 to 66.0)	5	2,311	55.7 (-23.9 to 84.2)
	105+	860	1,822	56.3 (46.9 to 64.0)	0	22	
		**SUS Pillar1 and 2 at least 1 day stay and respiratory code in primary diagnostic field**					
	**Interval (days)**	**cases**	**controls**	**VE (95% CI)**	**cases**	**controls**	**VE (95% CI)**
**unvaccinated**		**966**	**736**		**4,374**	**3,197**	
Dose 1	0-27	18	30	57.4 (-0.7 to 82)	79	200	55.1 (35.7 to 68.7)
	28+	146	274	52.3 (35.8 to 64.5)	623	3,522	74.7 (71.6 to 77.6)
Dose 2	0-13	1	7		41	1,373	82.8 (75.0 to 88.2)
	14-174	74	326	80.5 (72.2 to 86.3)	10,252	46,368	85.7 (84.7 to 86.6)
	175+	972	2,986	58.4 (51.0 to 64.7)	6,299	15,711	78.2 (76.6 to 79.8)
3rd dose	0-6	42	562	78.5 (66.8 to 86.1)	512	2,109	86.8 (84.8 to 88.5)
	7-13	44	637	82.2 (73.0 to 88.3)	401	2,200	88.0 (86.2 to 89.6)

	Interval (days)	cases	controls	VE (95% CI)	cases	controls	VE (95% CI)
	14-34	189	2,949	91.3 (89.1 to 93.0)	354	6,794	96.4 (95.8 to 96.9)
	35-69	892	8,390	88.9 (87.1 to 90.6)	208	9,008	95.7 (94.9 to 96.4)
	70-104	1,826	6,514	87.6 (85.6 to 89.3)	18	3,187	91.2 (85.2 to 94.8)
	105+	983	2,332	84.1 (81.2 to 86.5)	0	26	
		**SUS Pillar1 and 2 at least 2 days stay and respiratory code in primary diagnostic field**					
	**Interval (days)**	**cases**	**controls**	**VE (95% CI)**	**cases**	**controls**	**VE (95% CI)**
**unvaccinated**		**894**	**667**		**4,198**	**2,976**	
Dose 1	0-27	17	27	43.9 (-41 to 77.7)	73	191	59.2 (41.1 to 71.8)
	28+	134	248	53.4 (36.3 to 65.9)	593	3,263	74.5 (71.2 to 77.4)
Dose 2	0-13	0	5		40	1,253	81.8 (73.3 to 87.6)
	14-174	65	305	82.3 (74.3 to 87.8)	9,584	42,489	85.6 (84.6 to 86.6)
	175+	913	2,733	57.7 (49.6 to 64.4)	5,937	14,482	78.3 (76.6 to 79.9)
3rd dose	0-6	41	507	77.9 (65.3 to 85.9)	470	1,893	86.9 (84.8 to 88.6)
	7-13	39	595	84.7 (76.0 to 90.2)	362	2,024	88.6 (86.8 to 90.1)
	14-34	177	2,693	91.3 (89.1 to 93.1)	339	6,162	96.3 (95.7 to 96.8)
	35-69	801	7,616	89.3 (87.3 to 90.9)	194	8,199	95.7 (94.8 to 96.4)
	70-104	1,637	5,825	88.1 (86.1 to 89.9)	18	2,849	90.2 (83.5 to 94.3)
	105+	847	2,036	85.3 (82.4 to 87.6)	0	25	
		**SUS Pillar1 and 2 at least 3 days stay and respiratory code in primary diagnostic field**					
	**Interval (days)**	**cases**	**controls**	**VE (95% CI)**	**cases**	**controls**	**VE (95% CI)**
**unvaccinated**		**835**	**603**		**3,947**	**2,753**	
Dose 1	0-27	15	23	35.1 (-80.6 to 76.7)	70	166	55.0 (33.8 to 69.4)
	28+	120	232	57.6 (41.3 to 69.3)	559	3,000	74.2 (70.7 to 77.3)
Dose 2	0-13	0	4		36	1,152	82.7 (74.0 to 88.5)
	14-174	61	281	82.2 (73.7 to 87.9)	8,815	38,698	85.7 (84.7 to 86.7)
	175+	841	2,488	57.8 (49.4 to 64.9)	5,561	13,261	78.1 (76.3 to 79.7)
3rd dose	0-6	38	463	77.9 (64.7 to 86.2)	436	1,702	86.4 (84.2 to 88.3)
	7-13	38	552	84.5 (75.5 to 90.2)	335	1,831	88.3 (86.4 to 90.0)
	14-34	163	2,416	91.4 (89.0 to 93.2)	317	5,536	96.1 (95.5 to 96.6)
	35-69	715	6,772	89.5 (87.5 to 91.2)	179	7,331	95.6 (94.7 to 96.3)
	70-104	1,469	5,191	88.6 (86.5 to 90.3)	16	2,546	89.7 (82.1 to 94.1)
	105+	724	1,771	86.4 (83.6 to 88.7)	0	22	
		**SUS Pillar1 and 2 at least 2 days stay & oxygen with respiratory code in primary diagnostic field**					
	**Interval (days)**	**cases**	**controls**	**VE (95% CI)**	**cases**	**controls**	**VE (95% CI)**
**unvaccinated**		**138**	**41**		**920**	**163**	
Dose 1	0-27	2	1		18	6	
	28+	10	6	48.3 (-169.1 to 90)	71	124	82.3 (71.7 to 89.0)
Dose 2	0-13				6	62	88.1 (64.4 to 96.0)
	14-174	5	13	87.7 (50.9 to 96.9)	1,503	1,940	90.1 (87.3 to 92.2)
	175+	69	154	74.0 (47.6 to 87.1)	893	694	83.9 (78.8 to 87.8)

3rd dose	0-6	6	27	70.0 (-33.9 to 93.3)	69	106	93.5 (89.2 to 96.0)
	7-13	3	31	86.7 (-13.9 to 98.5)	48	102	91.6 (86.1 to 94.9)
	14-34	10	125	95.9 (89.0 to 98.4)	56	318	97.7 (96.3 to 98.5)
	35-69	70	398	93.9 (88.4 to 96.8)	39	446	96.7 (94.4 to 98.1)
	70-104	146	281	93.2 (87.5 to 96.2)	2	131	90.0 (43.4 to 98.2)
	105+	58	78	90.1 (79.7 to 95.2)			
		**SUS Pillar1 and 2 at least 2 days stay & either oxygen, ventilation or ICU with respiratory code in primary diagnostic field**					
	**Interval (days)**	**cases**	**controls**	**VE (95% CI)**	**cases**	**controls**	**VE (95% CI)**
**unvaccinated**		**215**	**70**		**1664**	**294**	
Dose 1	0-27	4	3		27	14	24.5 (-141.3 to 76.4)
	28+	16	16	78.3 (43.7 to 91.7)	131	269	86.7 (81.4 to 90.5)
Dose 2	0-13				10	112	89.8 (74.5 to 95.9)
	14-174	7	30	90.9 (72.6 to 97.0)	2570	3,491	90.5 (88.6 to 92.1)
	175+	108	249	73.4 (55.1 to 84.3)	1494	1,174	84.9 (81.5 to 87.7)
3rd dose	0-6	6	37	89.2 (63.1 to 96.8)	122	150	92.1 (88.4 to 94.6)
	7-13	4	57	94.7 (71.6 to 99.0)	92	184	92.3 (88.9 to 94.7)
	14-34	17	236	95.8 (91.3 to 97.9)	89	543	97.5 (96.5 to 98.3)
	35-69	115	638	92.8 (88.4 to 95.6)	61	692	96.1 (94.0 to 97.4)
	70-104	220	467	92.5 (88.1 to 95.2)	5	223	87.0 (58.6 to 95.9)
	105+	95	118	86.8 (77.1 to 92.3)			

Vaccine effectiveness estimates are adjusted using sex, index of multiple deprivation (quintile), ethnic group, care home residence status (for age 65+), geographic region (NHS region), period (calendar week of test), health and social care worker status (for age <65), clinical risk group status (for age<65), clinically extremely vulnerable, severely immunosuppressed, and previously testing positive

Table S11: Vaccine effectiveness by manufacturer against hospital admissions from emergency care (ECDS) within 14 days of the test date by the Omicron variant in symptomatic individuals 18 to 64 years of age

Manufacturer	Doses	Pillar 2 symptomatic with ECDS admission within 14 days				Pillar 2 symptomatic with respiratory coded ECDS admission within 14 days		
		**Interval (days)**	**cases**	**controls**	**VE (95% CI)**	**cases**	**controls**	**VE**
		**unvaccinated**	**643**	**127,803**		**171**	**127803**	
ChAdOx1-S	Dose 1	0-27	0	31		0	31	
		28+	50	9267	17.6 (-10.5 to 38.5)	14	9267	30.3 (-21.4 to 60.0)
	Dose 2	0-13	0	223		0	223	
		14-174	66	76181	38.5 (20.2 to 52.6)	15	76181	52.6 (18.7 to 72.4)
		175+	444	151244	31.6 (22.3 to 39.7)	102	151244	50.0 (35.4 to 61.3)
	3rd dose (any)	0-6	62	51422	58.2 (45.4 to 68.0)	12	51422	74.1 (52.9 to 85.8)
	3rd dose (BNT162b2)	7-13	25	37798	85.2 (77.9 to 90.1)	9	37798	83.0 (66.4 to 91.4)
		14-34	121	110195	84.1 (80.5 to 87.0)	25	110195	89.7 (84.1 to 93.3)
		35-69	278	114444	76.3 (72.3 to 79.6)	53	114444	87.5 (82.5 to 91.0)
		70+	161	32156	65.6 (58.5 to 71.6)	51	32156	73.4 (62.3 to 81.2)
		105+	52	4808	38.2 (16.0 to 54.5)	18	4808	
	3rd dose (mRNA-1273)	7-13	11	21600	88.1 (78.3 to 93.5)	0	21600	
		14-34	50	56829	88.3 (84.3 to 91.3)	5	56829	96.2 (90.7 to 98.4)
		35-69	83	43238	80.7 (75.5 to 84.8)	16	43238	88.1 (79.8 to 93.0)
		70+	15	5316	74.1 (56.3 to 84.7)	1	5316	
BNT162b2	Dose 1	0-27	25	8273	38.0 (7.3 to 58.5)	6	8273	41.7 (-32.0 to 74.3)
		28+	88	35531	44.9 (31.0 to 56.0)	12	35531	70.4 (46.8 to 83.6)
	Dose 2	0-13	13	6607	52.8 (18.1 to 72.8)	3	6607	56.8 (-35.8 to 86.2)
		14-174	340	321184	61.0 (55.3 to 65.9)	59	321184	71.7 (61.6 to 79.2)
		175+	207	60035	33.0 (21.3 to 42.9)	48	60035	48.0 (27.8 to 62.5)
	3rd dose (any)	0-6	41	43987	73.7 (63.8 to 80.9)	5	43987	87.2 (68.7 to 94.8)
	3rd dose (BNT162b2)	7-13	37	29861	77.5 (68.4 to 83.9)	4	29861	92.5 (76.4 to 97.6)
		14-34	84	77362	82.8 (78.4 to 86.3)	17	77362	87.4 (79.2 to 92.4)
		35-69	183	119375	78.6 (74.6 to 82.0)	41	119375	86.1 (80.1 to 90.2)
		70+	188	67343	72.5 (67.1 to 77.0)	46	67343	81.8 (74.0 to 87.3)
		105+	79	21177	67.7 (58.2 to 75.0)	19	21177	
	3rd dose (mRNA-1273)	7-13	8	12923	88.5 (76.8 to 94.3)	2	12923	88.4 (53.2 to 97.1)
		14-34	28	29200	87.3 (81.2 to 91.3)	5	29200	91.0 (78.1 to 96.3)
		35-69	32	23649	84.5 (77.9 to 89.2)	4	23649	93.3 (81.9 to 97.5)
		70+	11	2144	60.6 (27.7 to 78.5)	1	2144	0

Vaccine effectiveness estimates are adjusted using sex, index of multiple deprivation (quintile), ethnic group, care home residence status (for age 65+), geographic region (NHS region), period (calendar week of test), health and social care worker status (for age <65), clinical risk group status (for age<65), clinically extremely vulnerable, severely immunosuppressed, and previously testing positive

Table S12: Vaccine effectiveness by manufacturer against hospital admissions from emergency care (ECDS) within 14 days of the test date by the Omicron variant in symptomatic individuals 65 years of age and older

Manufacturer	Doses	Pillar 2 symptomatic with ECDS admission within 14 days				Pillar 2 symptomatic with respiratory coded ECDS admission within 14 days		
		Interval (days)	cases	controls	VE (95% CI)	cases	controls	VE (95% CI)
		unvaccinated	103	1,705		49	1,705	
ChAdOx1-S	Dose 1	0-27	0	2		0	2	
		28+	10	280		4	280	
	Dose 2	0-13	0	6		0	6	
		14-174	5	390	55.3 (-20.1 to 83.3)	2	390	69.9 (-38.4 to 93.5)
		175+	88	4,456	56.3 (39.6 to 68.3)	35	4,456	68.9 (49.7 to 80.8)
	3rd dose (any)	0-6	5	1,554	84.8 (61.1 to 94.1)	1	1,554	95.1 (62.8 to 99.4)
	3rd dose (BNT162b2)	7-13	3	1,872	93.0 (77.5 to 97.8)	1	1,872	95.5 (67.0 to 99.4)
		14-34	21	14,166	93.5 (89.3 to 96.0)	8	14,166	95.3 (89.7 to 97.8)
		35-69	137	35,872	92.7 (90.3 to 94.6)	47	35,872	95.1 (92.3 to 96.8)
		70+	154	14,449	91.0 (88.1 to 93.3)	60	14,449	93.3 (89.7 to 95.6)
		105+	71	2,602	82.9 (75.4 to 88.1)	30	2,602	85.1 (74.4 to 91.4)
	3rd dose (mRNA-1273)	7-13	0	811		0	811	
		14-34	2	3,472	98.1 (92.1 to 99.5)	1	3,472	98.1 (85.8 to 99.7)
		35-69	10	3,445	94.9 (90.0 to 97.4)	1	3,445	98.9 (92.1 to 99.9)
		70+	5	775	89.6 (73.8 to 95.9)	3	775	
BNT162b2	Dose 1	0-27	2	151	76.1 (-8.9 to 94.7)	1	151	
		28+	6	324	78.0 (47.7 to 90.8)	1	324	93 (47.8 to 99.1)
	Dose 2	0-13	0	22		0	22	
		14-174	8	569	85.8 (69.4 to 93.4)	3	569	90.4 (67.1 to 97.2)
		175+	28	1,792	75.6 (61.4 to 84.6)	7	1,792	89.5 (75.8 to 95.4)
	3rd dose (any)	0-6	4	569	72.7 (19.1 to 90.8)	1	569	
	3rd dose (BNT162b2)	7-13	2	882	90.1 (58.6 to 97.6)	1	882	
		14-34	4	6,805	97.4 (92.8 to 99.1)	0	6,805	
		35-69	113	26,699	91.7 (88.8 to 93.9)	51	26,699	92.8 (88.8 to 95.3)
		70+	260	18,863	90.2 (87.2 to 92.5)	102	18,863	92.7 (89.1 to 95)
		105+	113	4,379	88.0 (83.4 to 91.4)	48	4,379	90.3 (84.3 to 94.1)
	3rd dose (mRNA-1273)	7-13	1	241	89.0 (17.2 to 98.5)	0	241	
		14-34	4	1,076	91.2 (75.5 to 96.9)	1	1,076	95.9 (69.5 to 99.5)
		35-69	1	1,112		0	1,112	
		70+	0	276		0	276	

Table S13: Vaccine effectiveness using secondary care hospital admission data (SUS) by manufacturer for the Omicron variant in individuals 18-64 years of age

Manufacturer			SUS Pillar1 and 2 at least 2 days stay and respiratory code in primary diagnostic field			SUS Pillar1 and 2 at least 2 days stay & either O2, Vent or ICU and respiratory code in primary diagnostic field		
		Interval (days)	cases	controls	VE (95% CI)	cases	controls	VE (95% CI)
	Doses	unvaccinated	1016	696		227	87	
ChAdOx1-S	Dose 1	28+	88	153	48.5 (25.7 to 64.3)	12	23	82.8 (54.3 to 93.5)
	Dose 2					0	1	
		14-174	37	182	59.0 (31.9 to 75.3)	7	25	
		175+	406	877	53.0 (41.7 to 62.0)	55	140	81.9 (65.7 to 90.4)
	3rd dose (any)	0-6	21	120	78.0 (59.5 to 88.1)	4	14	90.4 (53.9 to 98.0)
	3rd dose (BNT162b2)	7-13	13	131	90.2 (78.1 to 95.6)	0	16	
		14-34	63	415	88.9 (83.8 to 92.4)	5	58	96.6 (88.7 to 99.0)
		35-69	184	632	83.9 (79.1 to 87.5)	19	88	93.2 (85.4 to 96.9)
		70+	151	327	82.2 (76.3 to 86.7)	20	38	90.6 (75.7 to 96.4)
		105+	61	67	69.0 (50.3 to 80.7)	7	6	
	3rd dose (mRNA-1273)	7-13	3	59	97.2 (86.1 to 99.4)	0	3	
		14-34	16	127	93.0 (86.4 to 96.4)	2	17	96.0 (64.9 to 99.5)
		35-69	32	123	89.2 (82.5 to 93.3)	2	10	95.1 (72.9 to 99.1)
		70+	13	30		0	3	
BNT162b2	Dose 1	0-27	24	21		4	3	
		28+	89	110	31.7 (-3.1 to 54.8)	10	8	
	Dose 2	0-13	5	12		0	2	
		14-174	89	225	73.8 (62.5 to 81.7)	5	18	88.7 (56.0 to 97.1)
		175+	108	272	65.1 (51.3 to 74.9)	10	32	82.3 (45.6 to 94.2)
	3rd dose (any)	0-6	9	27		0	3	
	3rd dose (BNT162b2)	7-13	4	50	85.2 (47.1 to 95.8)	0	8	
		14-34	44	168	79.7 (66.3 to 87.7)	1	24	99.5 (90.2 to 100.0)
		35-69	100	335	86.6 (81.3 to 90.4)	10	51	95.1 (86.7 to 98.2)
		70+	139	199	79.3 (71.3 to 85.0)	15	24	88.0 (67.1 to 95.6)
		105+	63	53	66.0 (44.5 to 79.2)	4	6	
	3rd dose (mRNA-1273)	7-13	2	9		0	1	
		14-34	10	50	94.3 (85.0 to 97.8)	1	8	
		35-69	13	38	89.8 (77.9 to 95.3)	0	5	
		70+	7	11		1	1	

Vaccine effectiveness estimates are adjusted using sex, index of multiple deprivation (quintile), ethnic group, care home residence status (for age 65+), geographic region (NHS region), period (calendar week of test), health and social care worker status (for age <65), clinical risk group status (for age<65), clinically extremely vulnerable, severely immunosuppressed, and previously testing positive

Table S14: Vaccine effectiveness using secondary care hospital admission data (SUS) by manufacturer for the Omicron variant in individuals 65 years of age and older

Manufacturer			SUS Pillar1 and 2 at least 2 days stay and respiratory code in primary diagnostic field			SUS Pillar1 and 2 at least 2 days stay & either oxygen, ventilation or ICU with respiratory code in primary diagnostic field		
		Interval (days)	cases	controls	VE (95% CI)	cases	controls	VE (95% CI)
	**Doses**	**unvaccinated**	**894**	**667**		**215**	**70**	
**ChAdOx1-S**	Dose 1	0-27	0	2		0	1	
		28+	80	164	48.1 (23.5 to 64.8)	11	11	77.3 (29.2 to 92.7)
	Dose 2	0-13	0	1				
		14-174	35	151	71.2 (50.0 to 83.4)	2	15	
		175+	640	1,815	53.1 (43.4 to 61.2)	70	171	71.4 (48.6 to 84.0)
	3rd dose (any)	0-6	30	378	76.7 (61.0 to 86.1)	4	28	92.1 (65.9 to 98.2)
	3rd dose (BNT162b2)	7-13	19	322	85.4 (73.4 to 92.0)	2	29	94.6 (36.2 to 99.5)
		14-34	96	1,536	91.3 (88.5 to 93.5)	12	144	95.0 (88.8 to 97.7)
		35-69	464	3,777	89.2 (87.1 to 91.0)	68	315	93.4 (88.8 to 96.2)
		70+	752	2,245	87.6 (85.2 to 89.6)	101	179	91.8 (86.4 to 95.1)
		105+	240	592	86.1 (82.5 to 88.9)	25	44	92.1 (83.8 to 96.2)
	3rd dose (mRNA-1273)	7-13	5	66		0	11	
		14-34	22	223	92.9 (87.7 to 95.9)	1	17	99.1 (85.8 to 99.9)
		35-69	38	244	92.7 (89.1 to 95.2)	6	25	96.3 (88.5 to 98.8)
		70+	19	94	91.8 (85.9 to 95.3)	1	7	
**BNT162b2**	Dose 1	0-27	13	24	54.8 (-25.3 to 83.7)	3	2	
		28+	53	83	59.5 (35.5 to 74.6)	5	5	
	Dose 2	0-13	0	4				
		14-174	29	150	87.6 (79.4 to 92.5)	4	15	88.9 (57.3 to 97.1)
		175+	273	918	65.4 (56.6 to 72.5)	38	78	76.7 (52.9 to 88.5)
	3rd dose (any)	0-6	11	129	80.6 (54.4 to 91.7)	2	9	
	3rd dose (BNT162b2)	7-13	13	161	86.4 (69.1 to 94.0)	2	14	93.2 (10.0 to 99.5)
		14-34	51	822	90.0 (85.4 to 93.2)	4	68	95.4 (80.9 to 98.9)
		35-69	275	3467	88.4 (85.7 to 90.6)	39	288	90.1 (82.1 to 94.6)
		70+	863	3450	88.4 (86.2 to 90.2)	117	278	92.8 (88.2 to 95.7)
		105+	607	1439	85.2 (82.1 to 87.7)	70	74	83.7 (70.6 to 91.0)
	3rd dose (mRNA-1273)	7-13	2	45	92.9 (50.2 to 99.0)	0	3	
		14-34	8	112	92.9 (83.0 to 97.1)	0	7	
		35-69	24	128	90.9 (84.8 to 94.5)	2	10	
		70+	3	41	97.3 (90.8 to 99.2)	1	3	

Vaccine effectiveness estimates are adjusted using sex, index of multiple deprivation (quintile), ethnic group, care home residence status (for age 65+), geographic region (NHS region), period (calendar week of test), health and social care worker status (for age <65), clinical risk group status (for age<65), clinically extremely vulnerable, severely immunosuppressed, and previously testing positive

Table S15: SUS ICD10 and OPCS code lists

**SUS Acute respiratory illness ICD10 code list**	
J04*	Acute laryngitis and tracheitis
J09*	Influenza due to identified avian influenza virus
J10*	Influenza with pneumonia, other influenza virus identified
J11*	Influenza with pneumonia, virus not identified
J12*	Viral pneumonia, not elsewhere classified
J13*	Pneumonia due to Streptococcus pneumoniae
J14*	Pneumonia due to Haemophilus influenzae
J15*	Bacterial pneumonia, not elsewhere classified
J16*	Pneumonia due to other infectious organisms, not elsewhere classified
J17*	Pneumonia in diseases classified elsewhere
J18*	Pneumonia, organism unspecified
J20*	Acute bronchitis
J21*	Acute bronchiolitis
J22*	Unspecified acute lower respiratory infection
J80*	ARDS (related to respiratory infection)
U071*	COVID-19, virus identified
U072*	COVID-19, Virus not identified
U04*	Severe acute respiratory syndrome [SARS]
**SUS OPCS-4 code list**	
X52*	Oxygen use
E85*	Ventilation support
E89*	Other respiratory support

Figure S1: Flow chart demonstrating the selection of data in the study

Data on all **negative Pillar 1 PCR tests** with an NHS number and date of birth from individuals with a sample date from 25 November 2020 to 17 February 2022 were extracted.

SUS linkage - restricted to tests with a respiratory ICD coded discharge diagnosis (any diagnosis field) where the sample was taken 1 days before and up to 2 days after the admission. Linkaged was on 21 February 2022.

Data on **all positive tests** regardless of symptom status (Pillar 1 (PCR) and Pillar 2 (PCR and LFT)) and **negative tests** (Pillar 2 (PCR)) from symptomatic individuals with a sample date from 25 November 2020 to 17 February 2022 were extracted.

Where participants had a positive test within +/- 14 days of another positive, the earliest PCR test was retained. Where tests were on the same day pillar 2 symptomatic tests were retained. Variant information was enriched using data from these other tests where it was missing. Other tests were then excluded.

Positive and negative tests within 90 days of a previous positive test were excluded.

Negative tests taken within 21 days after a positive test were excluded

Data were restricted to tests which could be linked to the vaccination record and had an NHS number

Linked on NHS number and DOB

ECDS linkage: an emergency admission (not injury) within 14 days of sample date (until day of admission). Linkage was on 22 February 2022.

SUS linkage: Pillar 2 tests-ICD respiratory discharge diagnosis (any diagnosis field) where the sample was taken 14 days before and up to 2 days after the day of admission. Pillar 1 tests- respiratory ICD coded discharge diagnosis where the sample was taken 1 days before and up to 2 days after the admission. Restricted to tests up to 2nd Feburary 2022 to account for delays in SUS data recording. Linkage was on 22 February 2022.

Only Samples from 26th April 2021 (Delta period onwards) retained

A previous negative test that led to a hospitalisation within the previous 21 days was excluded (done separatley)

A maximum of one negative test per person within each of the following approximate 3 month periods was selected at random: 26 April to 1 Aug 2021, 2 Aug 2021 to 21 Nov 2021, 22 Nov 2021- 2 February 2022.

Only tests taken until February 2nd 2022 were retained to allow sufficent time for hospitalisation

Those with unusual vaccinantion history dropped. This is i) Boosted before 13 Sep 2021 (date booster use started), ii) Boosted at <12 weeks of dose2, iii_) Four or more doses at the sample/onset date, iv) Gap between first 2 doses of <19 days, v) Mixed primary schedule at time of onset/boost, vi) Received any vaccine that is not ChAdOx1-S/BNT162b2/mRNA-1273, vii) boosted with ChAdOx1-S

Only those where variant deemed likely to be Delta or likely to be Omicron retained*

Dataset for Analysis

* Omicron is SGTF from 29 November 2021. Also from 10 January 2022 all cases if no sequencing/genotyping done. Delta is no SGTF from 26 April 2021 to 3 January 2022. Also all cases from 23 May-21 November 2021 if no SGTF/sequenced/genotype done. If sequencing is done then that result is used to classify the sample irrespective of S-gene status. Omicron is VOC-21NOV-01 or VUI-22JAN-01and Delta is VOC-21APR-02 or VUI-21OCT-01. If genotyping is done but no sequencing then this is used

Table S16: Unadjusted* vaccine effectiveness against different hospitalisation outcomes with Omicron by dose and interval (all vaccines combined)

		Pillar 2s ECDS (ECDS controls)	Pillar 2s ECDS respiratory coded (ECDS respiratory controls)	Pillar 1/ 2 SUS All respiratory coded 0 days	Pillar 1/ 2 SUS Not primary respiratory coded 1+ days	Pillar 1/ 2 SUS primary respiratory coded 1+ days	Pillar 1/ 2 SUS primary respiratory coded 2+ days	Pillar 1/ 2 SUS primary respiratory coded 3+ days	Pillar 1/ 2 SUS primary respiratory coded & oxygen 2+ days	Pillar 1/ 2 SUS primary respiratory coded & oxygen, ventilation, or ICU 2+ days
**Age 18-64**										
	**Interval**	**VE (95% CI)**	**VE (95% CI)**	**VE (95% CI)**	**VE (95% CI)**	**VE (95% CI)**	**VE (95% CI)**	**VE (95% CI)**	**VE (95% CI)**	**VE (95% CI)**
Dose 1	0-27	53.8 (22.2 to 72.5)	64.8 (-40.3 to 91.2)	4.6 (-124.2 to 59.4)	-13.1 (-96.4 to 34.9)	43.7 (-4.6 to 69.7)	36.8 (-26.3 to 68.4)	37.8 (-36.2 to 71.5)		
	28+	48.7 (33.4 to 60.5)	72.0 (47.3 to 85.2)	12.6 (-20.9 to 36.8)	23.6 (7.6 to 36.8)	46.9 (33.6 to 57.6)	47.6 (32.4 to 59.4)	53.0 (37.4 to 64.7)	85.7 (60.1 to 94.9)	80.0 (59.4 to 90.1)
Dose 2	0-13	47.5 (-13.4 to 75.6)	70.9 (-50.2 to 94.4)	34.2 (-63.7 to 73.6)	-37.9 (-214.4 to 39.5)	72.2 (26.3 to 89.5)	71.6 (9.8 to 91.1)	68.7 (-4.0 to 90.6)		
	14-174	56.0 (47.3 to 63.3)	75.7 (61.9 to 84.5)	15.1 (-8.2 to 33.3)	4.6 (-12.5 to 19.0)	56.2 (45.3 to 64.9)	57.6 (44.6 to 67.6)	66.0 (53.5 to 75.1)	54.6 (-86.3 to 89.0)	83.3 (60.5 to 92.9)
	175+	33.6 (21.1 to 44.1)	36.7 (2.3 to 59.0)	26.4 (8.8 to 40.5)	47.7 (40.5 to 54.0)	52.8 (45.0 to 59.5)	55.9 (47.6 to 62.9)	59.5 (50.8 to 66.7)	78.1 (55.1 to 89.3)	76.7 (62.2 to 85.7)
3rd dose	0-6	64.6 (53.4 to 73.1)	76.0 (52.1 to 87.9)	30.7 (-3.5 to 53.6)	26.1 (-6.5 to 48.6)	58.5 (37.1 to 72.6)	67.4 (46.7 to 80.1)	72.0 (52.5 to 83.5)	73.0 (-37.4 to 94.7)	84.7 (39.8 to 96.1)
	7-13	80.7 (74.5 to 85.4)	91.7 (84.1 to 95.7)	62.6 (45.5 to 74.4)	65.9 (53.3 to 75.0)	81.3 (72.4 to 87.3)	86.2 (77.4 to 91.5)	91.1 (83.4 to 95.2)		
	14-34	81.9 (78.2 to 85.0)	89.7 (83.4 to 93.5)	60.0 (49.6 to 68.2)	69.2 (64.0 to 73.7)	83.9 (80.3 to 86.9)	85.7 (81.8 to 88.7)	87.7 (83.9 to 90.7)	93.4 (81.2 to 97.7)	96.0 (91.0 to 98.2)
	35-69	70.9 (65.6 to 75.3)	79.3 (68.7 to 86.3)	32.7 (18.1 to 44.7)	76.1 (72.9 to 78.9)	79.5 (76.1 to 82.4)	82.6 (79.2 to 85.4)	85.2 (81.9 to 88.0)	94.1 (87.4 to 97.2)	93.5 (88.9 to 96.2)
	70-104	62.3 (54.5 to 68.8)	60.3 (36.7 to 75.0)	-62.3 (-102.2 to -30.2)	77.3 (73.6 to 80.5)	66.7 (60.9 to 71.6)	73.7 (68.3 to 78.3)	76.1 (70.3 to 80.7)	85.1 (67.3 to 93.2)	86.3 (76.1 to 92.2)
	105+	46.4 (29.0 to 59.6)	28.0 (-44.8 to 64.2)	-346.3 (-537.5 to -212.4)	76.5 (70.0 to 81.6)	38.9 (21.7 to 52.4)	45.9 (27.8 to 59.5)	52.3 (32.7 to 66.2)	70.3 (-14.7 to 92.3)	73.2 (30.3 to 89.7)

Age 65+										
	**Interval**	**VE (95% CI)**	**VE (95% CI)**	**VE (95% CI)**	**VE (95% CI)**	**VE (95% CI)**	**VE (95% CI)**	**VE (95% CI)**	**VE (95% CI)**	**VE (95% CI)**
Dose 1	0-27				-0.1 (-137.3 to 57.8)	43.7 (-18.9 to 73.3)	37.6 (-38.3 to 71.9)	37.8 (-48.1 to 73.9)		
	28+			51.6 (-14.1 to 79.5)	34.1 (8.3 to 52.7)	53.8 (39.1 to 64.9)	55.6 (40.6 to 66.8)	61.4 (47.7 to 71.4)	45.9 (-135.8 to 87.6)	75.4 (41.2 to 89.8)
	14-174	74.9 (40.2 to 89.5)	85.8 (33.9 to 97.0)	10.4 (-243.2 to 76.6)	64.9 (46.3 to 77.1)	77.8 (69.4 to 83.8)	80.0 (71.9 to 85.7)	80.4 (72.2 to 86.2)	85.5 (46.1 to 96.1)	90.9 (74.9 to 96.7)
	175+	60.8 (35.3 to 76.2)	65.3 (5.2 to 87.3)	43.3 (5.8 to 65.8)	24.3 (8.3 to 37.5)	59.0 (52.2 to 64.8)	59.5 (52.4 to 65.5)	60.1 (52.6 to 66.3)	72.2 (49.1 to 84.8)	72.9 (56.9 to 82.9)
3rd dose	0-6	82.5 (55.2 to 93.1)	94.9 (65.3 to 99.2)	81.6 (27.9 to 95.3)	45.5 (12.0 to 66.2)	81.0 (72.0 to 87.1)	80.6 (71.0 to 87.0)	80.8 (70.8 to 87.4)	81.6 (36.5 to 94.7)	89.5 (68.6 to 96.5)
	7-13	91.0 (74.7 to 96.8)	90.1 (22.8 to 98.7)	71.7 (10.6 to 91.0)	68.5 (51.6 to 79.5)	83.7 (76.3 to 88.8)	85.4 (78.3 to 90.1)	85.5 (78.2 to 90.3)	86.1 (35.3 to 97.0)	91.2 (69.9 to 97.4)
	14-34	93.0 (87.5 to 96.0)	97.3 (91.8 to 99.1)	77.5 (57.8 to 88.0)	73.1 (66.2 to 78.7)	91.5 (89.6 to 93.1)	91.8 (89.8 to 93.3)	92.0 (89.9 to 93.6)	96.0 (90.5 to 98.3)	96.1 (92.5 to 97.9)
	35-69	86.8 (79.6 to 91.5)	91.8 (80.6 to 96.6)	68.6 (50.8 to 80.0)	69.1 (63.2 to 74.1)	88.7 (86.9 to 90.2)	89.4 (87.6 to 90.9)	89.8 (88.0 to 91.3)	92.4 (86.9 to 95.6)	91.8 (87.5 to 94.6)
	70-104	81.7 (71.9 to 88.1)	88.3 (72.2 to 95.1)	42.9 (12.0 to 63.0)	63.3 (56.6 to 68.9)	86.3 (84.3 to 88.0)	87.1 (85.1 to 88.8)	87.7 (85.8 to 89.5)	90.1 (83.5 to 94.0)	89.9 (85.0 to 93.2)
	105+	65.7 (43.8 to 79.0)	72.4 (24.5 to 89.9)	18.6 (-28.5 to 48.4)	61.1 (53.4 to 67.5)	82.6 (79.8 to 85.0)	84.0 (81.2 to 86.3)	85.2 (82.5 to 87.5)	87.2 (76.4 to 93.0)	84.3 (74.9 to 90.2)

*adjusted for period only

1. Andrews N, Stowe J, Kirsebom F, Toffa S, Rickeard T, Gallagher E, et al. Covid-19 Vaccine Effectiveness against the Omicron (B.1.1.529) Variant. New England Journal of Medicine. 2022.
2. NHS England. National COVID-19 and Flu Vaccination Programmes [Available from: https://www.england.nhs.uk/contact-us/privacy-notice/national-flu-vaccination-programme/.
3. UK Health Security Agency. Emergency department: weekly bulletins for 2021 2021 [Available from: https://www.gov.uk/government/publications/emergency-department-weekly-bulletins-for-2021.
